# Injectable Chondroitin Sulfate Methacrylate Hydrogel Microspheres Co‐Loaded with GLPM Nanozyme, Dexamethasone, and Stem Cells for Synergistic Osteoarthritis Therapy

**DOI:** 10.1002/advs.202517083

**Published:** 2026-01-30

**Authors:** Xiaochen Feng, Ying Fang, Hongwei Yu, Yicheng Wang, Yunze Xu, Chunxiao Shi, Haike Xia, Ranjith Kumar Kankala, Aizheng Chen, Shibin Wang, Chaoping Fu

**Affiliations:** ^1^ Institute of Biomaterials and Tissue Engineering & Fujian Provincial Key Laboratory of Biochemical Technology Huaqiao University Xiamen Fujian China

**Keywords:** chondroitin sulfate, hydrogel microspheres, injection microspheres, manganese dioxide, osteoarthritis

## Abstract

Osteoarthritis (OA) is a degenerative joint disease characterized by cartilage degradation, chronic inflammation, and subchondral bone remodeling. Conventional intra‐articular therapies provide limited relief and fail to address its multifactorial pathogenesis. Here, we present an injectable hydrogel microsphere platform that integrates antioxidative, anti‐inflammatory, and regenerative functions for localized OA management. Uniform (∼125 µm) chondroitin sulfate methacrylate (ChSMA)‐based microspheres are fabricated via microfluidic photocross‐linking. Manganese dioxide nanoparticles provided catalytic reactive oxygen species (ROS) scavenging, while dexamethasone sodium phosphate enabled sustained release, reducing TNF‐α and IL‐6 levels by ∼30%. Bone marrow mesenchymal stem cells (BMSCs) are co‐delivered to promote cartilage repair. In vitro, the microspheres reduce intracellular ROS, induce M2 macrophage polarization, and suppress inflammatory cytokines by 60–70%, with IL‐10 levels increased by ∼90%. 3D co‐culture supports chondrocyte/BMSC viability and matrix production. In vivo, intra‐articular injection in a rat OA model markedly reduces cartilage erosion, decreases osteophyte volume by 80%, and improves subchondral bone microarchitecture. Histological staining confirms matrix restoration and structural preservation, with Osteoarthritis Research Society International (OARSI) scores reduced by 88%. Collectively, this injectable hydrogel microsphere system offers a minimally invasive and integrated strategy, simultaneously delivering antioxidative, anti‐inflammatory, and regenerative effects for comprehensive OA management.

## Graphical abstract

A multifunctional injectable microsphere system is fabricated via UV‐assisted microfluidic cross‐linking, integrating nanozyme‐based ROS scavenging, sustained dexamethasone release, and stem cell delivery. Upon IA injection, the microspheres adapt to joint stress, suppress inflammation, and promote cartilage regeneration, offering a minimally invasive and synergistic strategy for localized treatment of osteoarthritis.

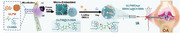

## 1 Introduction

Osteoarthritis (OA) is a highly prevalent degenerative joint disease that affects more than 500 million people worldwide, severely compromising mobility, quality of life, and imposing a growing socioeconomic burden [1, 2]. Current intra‐articular (IA) therapies, including corticosteroid injections and Visco supplementation, provide transient symptomatic relief but lack disease‐modifying effects, underscoring the urgent need for novel therapeutic strategies [3, 4].

Oxidative stress has emerged as a central driver of OA pathogenesis [5, 6]. Elevated levels of reactive oxygen species (ROS), including superoxide anions, hydroxyl radicals, and hydrogen peroxide, accumulate within the joint microenvironment, where they induce oxidative damage to cellular components, trigger chondrocyte apoptosis, and accelerate degradation of the cartilage extracellular matrix [7, 8]. Beyond direct tissue injury, excessive ROS activate pro‐inflammatory signaling cascades such as NF‐κB and MAPK, thereby amplifying synovial inflammation and driving progressive joint destruction [9, 10, 11]. This interplay between oxidative stress and inflammation contributes to a self‐perpetuating degenerative cycle, highlighting the critical role of redox imbalance in OA progression. Accordingly, targeting oxidative stress has gained increasing recognition as a promising therapeutic strategy to attenuate inflammation, preserve cartilage integrity, and restore joint homeostasis.

Consequently, modulating oxidative stress represents an attractive therapeutic avenue in OA management. In this regard, manganese dioxide (MnO_2_) has attracted significant interest due to its intrinsic catalase‐ and superoxide dismutase (SOD)‐like activities, enabling efficient scavenging of ROS and restoration of redox homeostasis [12, 13].To improve their biological safety and therapeutic efficacy, recent strategies have focused on green synthesis approaches employing natural biomolecules. Among these, Ganoderma lucidum polysaccharides (GLPs), known for their intrinsic antioxidant and immunomodulatory properties, have been widely explored as both a reducing and stabilizing agent [14]. Notably, the reduction of potassium permanganate using GLPs has been shown to produce MnO_2_ nanoparticles with improved biocompatibility and enhanced antioxidant capacity, offering a synergistic approach that combines nanozyme activity with natural bioactives [15, 16, 17].

Despite the crucial role of oxidative stress in OA, effective therapy must also address the limited regenerative capacity of articular cartilage [18, 19]. Bone marrow‐derived mesenchymal stem cells (BMSCs) and chondroitin sulfate (ChS) have been widely studied due to their chondrogenic potential, immunomodulatory effects, and matrix‐supporting functions [20, 21, 22, 23, 24, 25, 26]. However, their IA application suffers from low cell viability, poor joint retention, and insufficient engraftment, limiting their long‐term therapeutic efficacy [27, 28].

To overcome these challenges, hydrogel‐based IA delivery systems have gained attention for their ability to provide localized, sustained release while mimicking the native extracellular matrix [29, 30, 31, 32]. When engineered as injectable microspheres, hydrogels exhibit improved injectability, enhanced joint residence time, and uniform IA distribution. Microfluidic fabrication further allows precise control over microsphere size and structure [33, 34], while methacrylate‐based photo‐cross‐linking imparts tunable mechanical stability and degradation behavior compatible with IA environments [17, 35].

Importantly, recent studies have highlighted the pivotal role of immune modulation in OA pathophysiology, particularly the polarization of macrophages from the pro‐inflammatory M1 phenotype to the anti‐inflammatory M2 phenotype [36]. M2‐polarized macrophages secrete anti‐inflammatory cytokines and growth factors that facilitate cartilage repair and matrix remodeling [37]. Furthermore, hypoxia‐inducible factor‐1α (HIF‐1α) has emerged as a key regulator in this context, promoting M2 polarization and enhancing the regenerative microenvironment under hypoxic joint conditions [38]. Therefore, strategies capable of activating the HIF‐1α pathway and promoting M2 macrophage polarization hold considerable promise for restoring joint homeostasis in OA. Alongside immune modulation, stem cell–based therapies, particularly those using BMSCs, have attracted extensive interest due to their chondrogenic potential and secretion of trophic, immunoregulatory factors that support tissue repair [39]. Despite these biological advantages, free BMSCs delivered via intra‐articular injection exhibit poor retention, rapid clearance, and markedly reduced viability in the inflamed, oxidative OA microenvironment [40]. These limitations result in inconsistent therapeutic outcomes and undermine the long‐term regenerative capacity of stem cell therapy.

**FIGURE 1:**
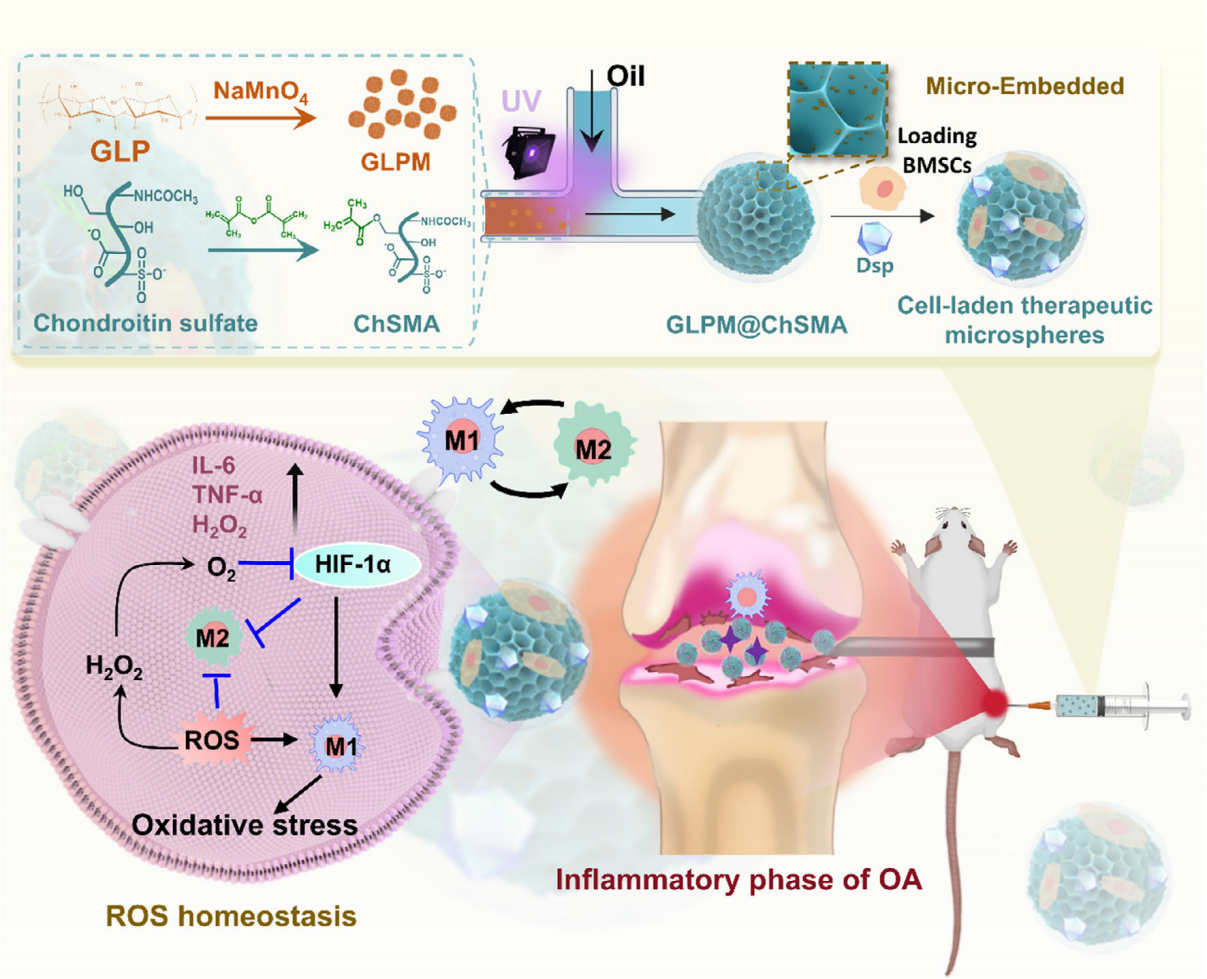
Schematic diagram of cell‐laden therapeutic microspheres for OA treatment.

In this work, we developed a multifunctional injectable hydrogel microsphere platform for the IA treatment of OA, integrating ROS scavenging, anti‐inflammatory drug delivery, and stem cell‐based cartilage regeneration (Figure 1). The microspheres were fabricated using chondroitin sulfate methacrylate (ChSMA) as a photocrosslinkable matrix via a UV‐assisted microfluidic process, ensuring precise control over particle size and internal structure. GLP‐reduced MnO_2_ nanoparticles (GLPM) were incorporated to provide antioxidative nanozyme activity, while dexamethasone sodium phosphate (Dsp) was included for sustained anti‐inflammatory effects. Moreover, BMSCs were co‐encapsulated to enhance cartilage repair through paracrine signaling and extracellular matrix remodeling. This platform is designed to respond to OA‐associated inflammation by scavenging ROS, modulating immune signaling via HIF‐1α stabilization and M2 macrophage polarization, and enabling synchronized degradation and spatiotemporal therapeutic release. Through the combined action of antioxidative nanozymes, sustained anti‐inflammatory drug delivery, and stem cell‐mediated regeneration, it offers a comprehensive strategy to mitigate oxidative stress, suppress inflammation, and promote cartilage repair.

## 2 Results and Discussion

### 2.1 Redox‐Synthesized GLPM Nanoparticles Exhibit Controlled Structure and ROS Scavenging Activity

**FIGURE 2:**
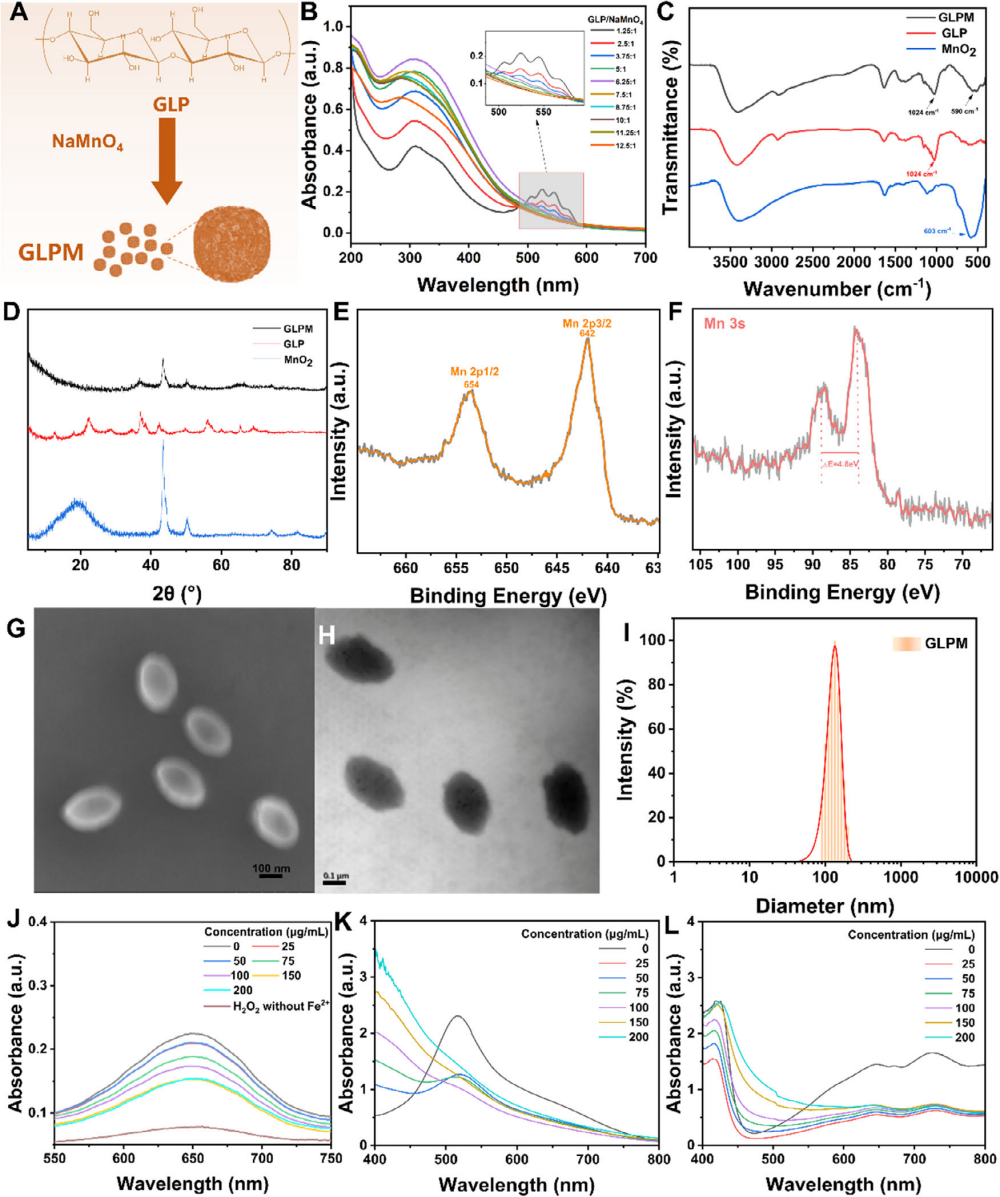
Preparation and compositional analysis of GLPM nanoparticles. (A),(B) UV–vis absorption spectra of reaction mixtures prepared with varying mass ratios of GLP and NaMnO_4_ after 24 h of reaction. (C) FTIR spectra of GLPM, GLP, and MnO_2_ powders. (D) XRD patterns of GLPM, GLP, and MnO_2_. XPS spectra of GLPM nanoparticles, showing (E) Mn 2p and (F) Mn 3s binding energies. (G) SEM image of GLPM nanoparticles. (H) TEM image of GLPM nanoparticles. (I) DLS analysis depicting particle size distribution of GLPM. (J) UV–vis spectra of the reaction solution after 10 min of reaction between GLPM and TMB. (K) UV–vis spectra showing changes after 30 min of reaction between various concentrations of GLPM and DPPH· radicals. (L) UV–vis spectra illustrating changes after 10 min of reaction between various concentrations of GLPM and ABTS·^+^ radicals.

As illustrated in Figure 1A, the GLPM nanoparticles were synthesized via a redox reaction between Ganoderma lucidum polysaccharide (GLP) and KMnO_4_, forming Mn‐based nanostructures with uniform morphology and antioxidant activity (Figure 2A). The GLPM nanoparticles were evaluated by ultraviolet‐visible (UV–vis) spectroscopy. As shown in Figure 2B, samples with GLP: NaMnO_4_ mass ratios below 7.5:1 displayed persistent absorption peaks between 480 and 600 nm, characteristic of unreacted Mn (VII) species. In contrast, formulations with higher GLP content yielded no detectable peaks in this region, indicating complete reduction of NaMnO_4_. A mass ratio of ≥7.5:1 was thus selected for subsequent synthesis to ensure reaction completion and purity of MnO_2_ nanoparticles.

Fourier transform infrared (FTIR) spectroscopy further verified the coordination between MnO_2_ and GLP. A redshifted Mn‐O vibration peak from 603 cm^−1^ in pure MnO_2_ to 590 cm^−1^ in GLPM suggested the formation of Mn‐O···OH or Mn‐O···COOH coordination bonds, likely due to interactions between GLP's hydroxyl and carboxyl groups and surface Mn atoms (Figure 2C). X‐ray diffraction (XRD) analysis revealed distinct peaks for MnO_2_ at 22°, 37°, and 64°, confirming its crystalline structure, while GLP and GLPM showed broad, low‐intensity peaks, consistent with their partially amorphous or disordered nature (Figure 2D).

X‐ray Photoelectron Spectroscopy (XPS) was employed to identify the oxidation state of manganese in the final GLPM product. The narrow Mn 2p_3/2_ peak at 642 eV and the Mn 3s multiplet splitting pattern were consistent with Mn (IV), confirming that the reduction process yielded MnO_2_ rather than lower‐valent manganese oxides (Figure 2E,F) [41].

Figure S1 presents the thermogravimetric analysis (TGA) curve of GLPM. An initial weight loss observed between 0 and 100°C corresponds to the evaporation of residual moisture. The subsequent weight reduction from 100 to 300°C is attributed to the thermal decomposition of GLP. Beyond 300°C, the weight remains relatively stable, indicating the formation of thermally stable residues.

Electron microscopy revealed morphological features consistent with nanoscale assembly. Scanning electron microscopy (SEM) showed uniform ellipsoidal particles with average diameters of approximately 120 nm, which was further corroborated by Transmission electron microscopy (TEM) imaging (Figure 2G,H). These findings validate the successful formation of redox‐synthesized GLPM nanoparticles with controlled size and homogeneity. Dynamic light scattering (DLS) analysis further confirmed a narrow size distribution with a mean hydrodynamic diameter of ∼130 nm, indicating excellent dispersity and structural homogeneity (Figure 2I).

The stability of nanoparticles is closely linked to their biocompatibility in vivo. Nanoparticles with good colloidal stability are less likely to induce potential toxicity or trigger immune responses, thereby improving their safety profile for clinical applications. Therefore, the stability of GLPM was systematically investigated. As shown in Figure S2A, GLPM remained well‐dispersed without noticeable aggregation or precipitation when incubated in ddH_2_O, phosphate‐buffered saline (PBS), and Dulbecco's Modified Eagle Medium (DMEM) over a period of seven days. Moreover, the DLS measurements presented in Figure S2B confirmed that the particle size of GLPM remained stable throughout the seven‐day observation period, indicating excellent colloidal stability in various physiological media.

Under mildly acidic conditions, ROS can oxidize colorless tetramethylbenzidine (TMB) into a blue oxidized form (OX‐TMB), which exhibits a strong absorption peak at 652 nm. In this study, a Fenton reaction system was established using an iron‐based catalyst and H_2_O_2_ to hydroxyl radicals (·OH), thereby simulating a ROS‐rich oxidative stress environment. The TMB assay was employed to assess the ROS‐scavenging capability of GLPM nanoparticles. As shown in Figure 2J and Figure S3A,B, the addition of GLPM led to a visible fading of the blue color, accompanied by a progressive decrease in absorbance at 652 nm with increasing GLPM concentration. Moreover, extending the reaction time resulted in a further reduction of absorbance, which plateaued after approximately 30 min (Figure S3C). These results indicate that GLPM exhibits efficient ·OH scavenging activity in a dose‐and time‐dependent manner.

In addition to ROS, reactive nitrogen species (RNS) play a crucial role in the inflammatory microenvironment of OA, promoting synovial inflammation and cartilage matrix degradation [42, 43]. The antioxidant properties of GLPM were further examined using the 1,1‐Diphenyl‐2‐picrylhydrazyl (DPPH·) assay, wherein DPPH·radicals, stabilized by resonance over three aromatic rings, exhibit a strong absorption band at approximately 520 nm and impart a deep purple color to the solution. As GLPM concentration increased, a notable decline in absorbance at 517 nm was observed (Figure 2K), with the peak nearly disappearing at 200 µg/mL, indicating that GLPM effectively neutralized DPPH· radicals in a concentration‐dependent manner.

Similarly, the ABTS assay was employed to assess the scavenging activity of GLPM against ABTS^+^· radicals. Under oxidative conditions, 2,2'‐azino‐bis(3‐ethylbenzothiazoline‐6‐sulfonic acid) (ABTS) is converted into a stable blue‐green radical cation ABTS^+^·, which exhibits a prominent absorption peak at 405 nm (Figure 2L). Upon treatment with increasing concentrations of GLPM, a marked decrease in absorbance at 405 nm was detected, corresponding to suppression of ABTS^+^·formation and demonstrating the strong radical‐scavenging capacity of GLPM. In addition, the nanozyme behavior of MnO_2_‐based systems has been rigorously validated in our previous work through full Michaelis–Menten kinetic analyses, confirming characteristic peroxidase‐like catalytic activity; given the identical MnO_2_ redox framework, GLPM is expected to exhibit comparable enzyme‐like catalytic mechanisms, providing mechanistic support for its broad‐spectrum antioxidant behavior [44].

Taken together, the results from the TMB, DPPH, and ABTS^+^·assays consistently confirm that GLPM possesses robust antioxidant activity, capable of scavenging both ROS and RNS in vitro. This broad‐spectrum radical scavenging suggests that GLPM could function as a nanozyme, contributing to the modulation of oxidative stress within osteoarthritic joints. Such activity holds therapeutic potential for alleviating inflammatory damage and slowing cartilage degeneration associated with OA progression.

### 2.2 Synthesis and Characterization of Photocrosslinkable ChSMA Microspheres

**FIGURE 3:**
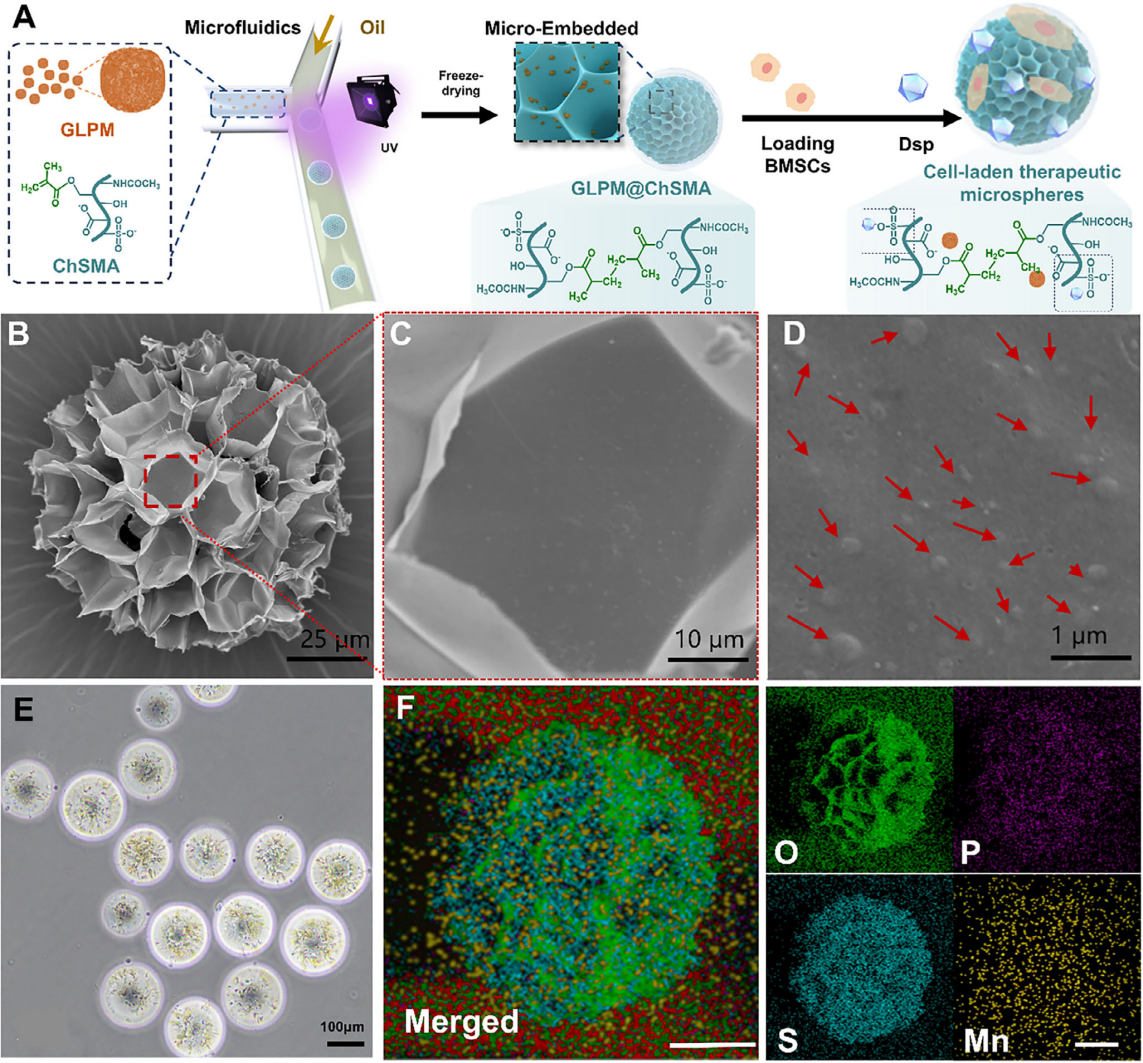
Synthesis and characterization of ChSMA and morphological analysis of hydrogel microspheres. (A) Schematic illustration of the synthetic route for microspheres. (B–D) Morphology and size characterization of GLPM@ChSMA hydrogel microspheres: SEM images at 40 × (B) and 100 × magnification (C), and particle size distribution analyzed using ImageJ software (D). (E) optical microscopy images of GLPM@ChSMA microspheres at different magnifications. (F) SEM image and EDS elemental mapping of GLPM/Dsp@ChSMA microspheres showing the distribution of oxygen (O), phosphorus (P), sulfur (S), and manganese (Mn). (Scale bar: 50 µm).

To engineer an injectable and bioresponsive platform for IA delivery, Chs was functionalized with methacrylate groups to yield photocrosslinkable ChSMA (Figure 3A). The methacrylation process was confirmed via proton nuclear magnetic resonance spectroscopy (^1^H NMR). As shown in Figure S4, the appearance of characteristic vinyl proton peaks at δ = 5.65 and 6.10 ppm, along with the methyl proton peak at δ = 1.84 ppm, validated successful modification. These peaks were absent in unmodified ChS, indicating the effective grafting of methacrylate functionalities onto the polymer backbone. Quantitative NMR analysis further determined that the methacrylation degree reached 21.8%, providing a clear and reproducible benchmark for the extent of functionalization.

SEM further revealed that the microspheres exhibited a highly porous architecture with interconnected networks (Figure 3B). Statistical analysis indicated an average particle diameter of ∼125 µm and an average pore size of ∼20 µm. Statistical analysis indicated an average particle diameter of ∼125 µm and an average pore size of ∼20 µm, which are advantageous for nutrient diffusion, therapeutic loading, and cell adhesion [45, 46, 47]. As shown in Figure 3D, high‐magnification SEM images also revealed the presence of uniformly distributed ellipsoidal nanoparticles embedded within the microsphere surface, consistent with the size and morphology of previously characterized GLPM nanoparticles.

Using a UV‐assisted microfluidic approach, GLPM/Dsp‐loaded ChSMA microspheres were fabricated with uniform morphology and tunable size. Optical microscopy images demonstrated consistent spherical geometry (Figure 3E). ImageJ analysis quantified the average microsphere diameter to be approximately 111.86 ± 11.11 µm (Figure S5), a dimension well‐suited for IA retention and injectability.

To confirm the successful encapsulation of therapeutic agents, energy‐dispersive X‐ray spectroscopy (EDS) mapping was conducted. As shown in Figure 3F, both phosphorus (from Dsp) and manganese (from GLPM) were uniformly distributed throughout the microsphere matrix, indicating effective co‐loading of both nanozyme and anti‐inflammatory drug components.

The multiscale structural integration of antioxidant GLPM nanoparticles, sustained‐release Dsp, and a cell‐adhesive ChSMA matrix offers a robust platform for dual‐modality OA therapy. Furthermore, the porous hydrogel microarchitecture recapitulates extracellular matrix (ECM) characteristics, providing a suitable scaffold for subsequent stem cell encapsulation and delivery.

### 2.3 Mechanical Robustness and Lubrication Performance of GLPM/Dsp@ChSMA Microspheres

**FIGURE 4:**
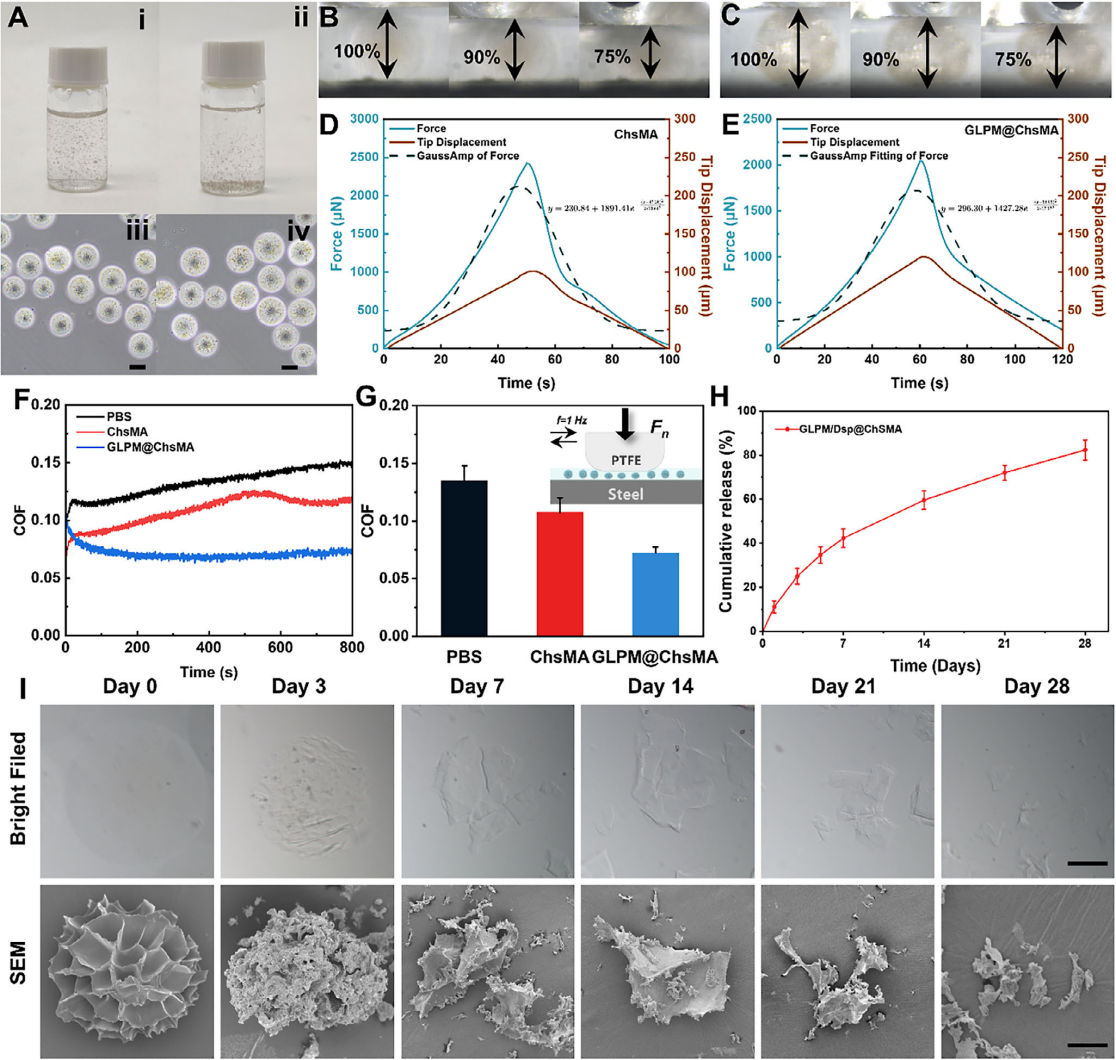
Evaluation of the injectability and physicochemical properties of GLPM/Dsp@ChSMA hydrogel microspheres. (A) Morphological comparison of microspheres before and after injection through a 25 G needle: (i, ii) macroscopic images and (iii, iv) microscopic images pre‐and post‐injection (scale bar: 100 µm). (B),(C) Optical images of microspheres under compressive deformation: (B) ChSMA microspheres and (C) GLPM@ChSMA microspheres. (D),(E) Force‐displacement curves of microspheres under compression: (D) ChSMA and (E) GLPM@ChSMA microspheres. (F) COF‐time plots and (G) COF histograms for PBS, ChSMA, and GLPM@ChSMA microspheres in PBS (5 mg/mL) under the loading of 12 N. (H) Cumulative release of Dsp from microspheres in the presence of collagenase II (2 U/mL, 37 °C). (I) Morphology of microspheres after 28 days of degradation, shown by optical microscopy (top) and SEM (bottom) (scale bar: 100 µm).

To validate the structural integrity of microspheres during administration, morphological assessments were conducted before and after injection through a 25 G needle. As shown in Figure 4A, the microspheres maintained their spherical morphology without visible surface cracking or deformation post‐injection, confirming their ability to endure shear stress and mechanical extrusion, crucial for minimally invasive IA delivery.

The compressive deformation behaviors of ChSMA and GLPM@ChSMA microspheres were further investigated to assess mechanical strength and structural resilience. As depicted in Figure 4B,C, both types of microspheres demonstrated elastic deformation from 100% to 75% of their original height under compression. Figure 4D shows that ChSMA microspheres reached a peak force of 1891.41 µN with a Gaussian fitting curve indicating brittle failure following elastic loading. In contrast, Figure 4E reveals that GLPM@ChSMA microspheres exhibited a lower peak force (1427.28 µN) but a broader Gaussian profile, reflecting improved elasticity and energy dissipation capacity. These results indicate that GLPM loading enhances mechanical adaptability and reduces brittleness, facilitating better tolerance to injection and IA mechanical stress. Given that osteophyte formation is highly dependent on joint instability and abnormal mechanical loading, conditions not reproducible in static in vitro culture, the improved elasticity and hydration‐mediated lubrication of GLPM@ChSMA microspheres (Figure 4F,G) provide an in vivo mechanical protective effect that can reduce aberrant bone overgrowth. By lowering joint friction and mitigating cartilage wear, the microspheres indirectly suppress mechanosensitive osteophyte development while promoting normalization of subchondral bone remodeling [48, 49].

Tribological testing using a standard steel‐PTFE friction pair in reciprocating mode (UMT‐2) further revealed enhanced lubrication performance. As shown in Figure 4F,G, GLPM@ChSMA microspheres significantly reduced the coefficient of friction (COF) compared to PBS or ChSMA alone, with a ∼46.7% decrease (from 0.135 to 0.072). This improvement is attributed to the microspheres’ elasticity and rolling behavior, which distribute mechanical loads more evenly and reduce shear stress at the articulating interface.

### 2.4 Inflammation‐Responsive Degradation and Sustained Drug Release Behavior

To simulate different joint environments, GLPM/Dsp@ChSMA microspheres were incubated in physiological (PBS, pH 7.4), enzymatic (collagenase II), and inflammatory (H_2_O_2_, pH 6.5) conditions. As shown in Figure S6, degradation was minimal in physiological PBS but accelerated significantly under oxidative stress, with nearly complete degradation by day 28. This rapid breakdown is attributed to the ROS‐scavenging activity of GLPM nanozymes, highlighting their inflammation‐responsiveness. Mechanistically, this is due to the redox reaction between GLPM and environmental H_2_O_2_, which reduces the MnO2 nanozymes into soluble Mn2+ ions and leads to the collapse of the nanozyme framework. As these MnO2 structures are micro‐embedded within the hydrogel matrix, their dissolution disrupts the internal architecture of the microspheres, thereby accelerating matrix degradation under inflammatory conditions [50, 51]. In enzymatic conditions, degradation proceeded more gradually, demonstrating controlled biodegradability appropriate for OA therapy timelines.

Drug release kinetics assessed by UV–vis absorbance showed a sustained release profile over 28 days (Figure 4H), with ∼80% cumulative release of Dsp. A mild burst release occurred in the first 24 h, followed by a diffusion‐dominated sustained release phase. The hydrogel network and tortuous porous architecture effectively regulated drug diffusion.

Swelling behavior (Figure S7) revealed rapid water uptake within 10 min (swelling ratio ∼1300%) and equilibrium (∼2400%) after 6 h. This high swelling capacity, driven by hydrophilic functional groups and porous microstructure, facilitates drug transport and matrix hydration.

Finally, SEM analysis (Figure 4I) showed progressive surface roughening and pore collapse during degradation, which enhanced surface area and drug release kinetics.

Collectively, these results confirm that GLPM/Dsp@ChSMA microspheres exhibit inflammation‐responsive degradation, sustained drug release, high swelling capacity, and structural evolvability, features that together enable long‐term retention, controlled therapeutic delivery, and bioadaptability within the OA joint microenvironment. A comparative overview with recently reported nanozyme‐loaded microsphere systems further underscores the distinct advantages of this platform in terms of multifunctional integration (Table S1).

### 2.5 Biocompatibility and Antioxidant Efficacy in Cellular Models

The biocompatibility of GLPM nanoparticles was first assessed by evaluating hemolytic activity, an essential safety parameter given the potential entry of nanoparticles into systemic circulation via synovial vasculature or lymphatics under inflammatory conditions in OA. As shown in Figure S8A, GLPM exhibited minimal hemolytic activity across tested concentrations, and Figure S8B confirmed that all microsphere formulations maintained hemolysis rates well within acceptable safety limits.

Cytocompatibility studies further confirmed the low toxicity profile of both GLPM and GLPM/Dsp@ChSMA microspheres. CCK‐8 assays showed that GLPM nanoparticles exerted negligible effects on the viability of rat chondrocytes and RAW 264.7 macrophages at concentrations up to 5 µg/mL over 48 h (Figure S9). Higher concentrations (10–50 µg/mL) induced only modest, time‐dependent reductions in cell viability (∼10–20%), suggesting good tolerance at therapeutic doses with minimal cytotoxicity risk during short‐term applications.

Calcein‐AM/PI staining provided visual confirmation of cell viability. Following co‐culture with GLPM at concentrations ranging from 0.5 to 50 µg/mL for 24 or 48 h, rat chondrocytes exhibited predominantly green fluorescence with minimal red fluorescence (Figure S10A). Quantification of fluorescence‐positive areas showed that live‐cell (Calcein‐AM) signals overwhelmingly dominated, with only a very small proportion of PI‐positive areas, confirming negligible cytotoxicity. Similarly, extracts from different microsphere formulations did not induce significant cell death over 1–3 days, consistent with CCK‐8 results (Figure S10B). Fluorescence quantification also demonstrated consistently high live/dead area ratios, supporting their excellent cytocompatibility.

**FIGURE 5:**
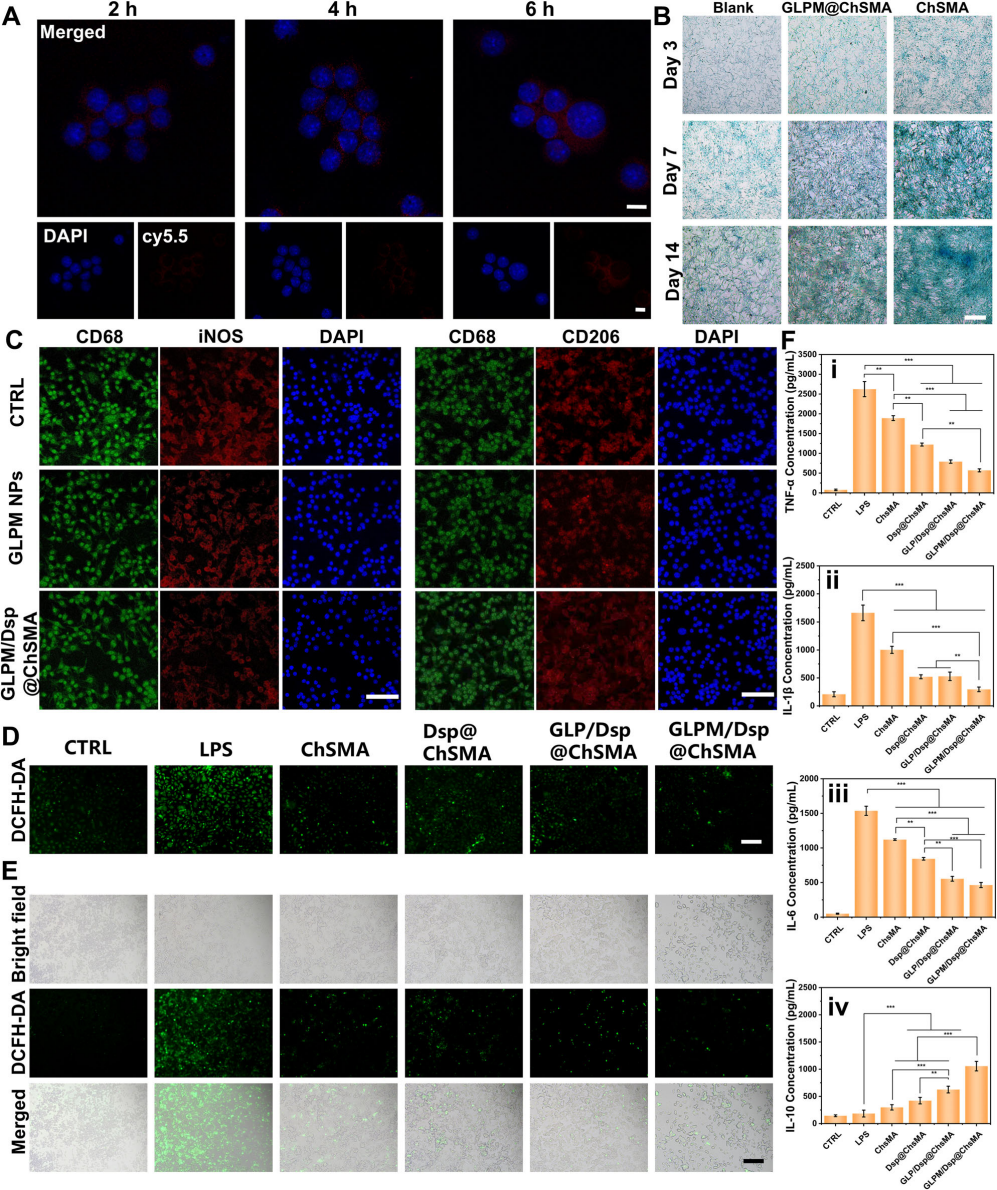
In vitro evaluation of cytocompatibility, cellular uptake, macrophage polarization, ROS scavenging, and inflammatory cytokine modulation by GLPM NPs and GLPM/Dsp@ChSMA hydrogel microspheres. (A) Cellular uptake of Cy5.5‐labeled GLPM nanoparticles by RAW 264.7 cells after 2, 4, and 6 h of incubation, visualized by confocal laser scanning microscopy (CLSM) (scale bar: 20 µm). (B) Alcian blue staining of chondrocytes cultured with Blank, GLPM@ChSMA, or ChSMA microspheres for 3, 7, and 14 days (scale bar: 500 µm). (C) Immunofluorescence images showing macrophage polarization profiles in RAW 264.7 cells after treatment with GLPM NPs and GLPM/Dsp@ChSMA microspheres. M1 macrophages were identified by co‐staining for CD68 and iNOS, and M2 macrophages by CD68 and CD206 markers (scale bar: 50 µm). (D) Intracellular ROS scavenging effect of different microsphere formulations in LPS‐induced rat chondrocytes. (E) Fluorescence images depicting ROS levels in LPS‐stimulated RAW 264.7 cells following treatment with different microspheres (scale bar: 200 µm). (F) Effects of various microspheres on inflammatory cytokine secretion in LPS‐induced RAW 264.7 cells, showing inhibition of pro‐inflammatory cytokines (i) TNF‐α, (ii) IL‐1β, and (iii) IL‐6, and upregulation of the anti‐inflammatory cytokine (iv) IL‐10 (*n* = 3; ^*^ *p* < 0.05, ^**^ *p* < 0.01, ^***^ *p* < 0.001).

Cellular internalization studies using Cy5.5‐labeled GLPM nanoparticles revealed time‐dependent uptake by RAW 264.7, with increasing fluorescence intensity observed between 2 and 6 h (Figure 5A). The punctate cytoplasmic localization suggests endosomal or lysosomal accumulation, supporting the potential for intracellular ROS scavenging.

Consistent with the requirement for cartilage matrix restoration in OA, microsphere‐mediated chondrogenic differentiation was further assessed using Alcian blue staining. As shown in Figure 5B, chondrocytes cultured with GLPM@ChSMA microspheres exhibited markedly enhanced deposition of sulfated glycosaminoglycans (sGAGs) over 3, 7, and 14 days compared with both the Blank and ChSMA‐only groups. The progressive deepening of blue staining indicates sustained extracellular matrix production, suggesting that the microenvironment provided by GLPM@ChSMA supports chondrocyte phenotype maintenance and promotes cartilage‐like matrix synthesis. In contrast, the blank control displayed limited sGAG accumulation, reflecting the lack of structural or biochemical cues. These results confirm that the hydrogel microspheres not only possess immunomodulatory and antioxidative functions but also contribute to the restoration of cartilage‐specific matrix, thereby complementing their therapeutic role in OA.

To explore the immunomodulatory impact of the microspheres, lipopolysaccharide (LPS)‐stimulated RAW 264.7 cells were treated with microsphere extracts, and macrophage polarization was assessed via immunofluorescence. As shown in Figure 5C, pro‐inflammatory M1 polarization was evident in LPS‐only controls (high iNOS expression), while treatment with GLPM/Dsp@ChSMA extracts significantly reduced iNOS fluorescence and upregulated CD206, a marker of anti‐inflammatory M2 macrophages [52]. These results suggest that GLPM‐containing microspheres not only scavenge intracellular ROS but also actively shift macrophage phenotypes toward a pro‐regenerative M2 state [53].

Intracellular ROS scavenging was further validated by DCF‐DA staining. LPS‐stimulated chondrocytes displayed intense green fluorescence, reflecting elevated ROS levels. Treatment with GLPM/Dsp@ChSMA microspheres markedly diminished fluorescence intensity, outperforming ChSMA and GLP/Dsp@ChSMA controls (Figure 5D,E; Figure S11). This superior antioxidant capacity is attributed to the nanozyme‐like catalytic activity of MnO_2_ within the GLPM nanoparticles.

Finally, enzyme‐linked immunosorbent assay (ELISA) analyses demonstrated that treatment with GLPM/Dsp@ChSMA microspheres significantly downregulated pro‐inflammatory cytokines TNF‐α, IL‐6, and IL‐1β (about 70%) while upregulating anti‐inflammatory IL‐10 (about 90%) in LPS‐stimulated RAW 264.7 cells (Figure 5F; Figure S12). Among all tested formulations, GLPM/Dsp@ChSMA achieved the strongest suppression of inflammatory markers, indicating synergistic benefits from ROS scavenging and immunomodulation.

Collectively, these results establish that GLPM/Dsp@ChSMA microspheres exhibit excellent biocompatibility, potent antioxidant activity, and significant immunomodulatory effects in vitro, underscoring their potential for mitigating inflammation and promoting cartilage repair in OA.

### 2.6 3D Cell Compatibility and Stem Cell Loading Capacity of Microspheres

**FIGURE 6:**
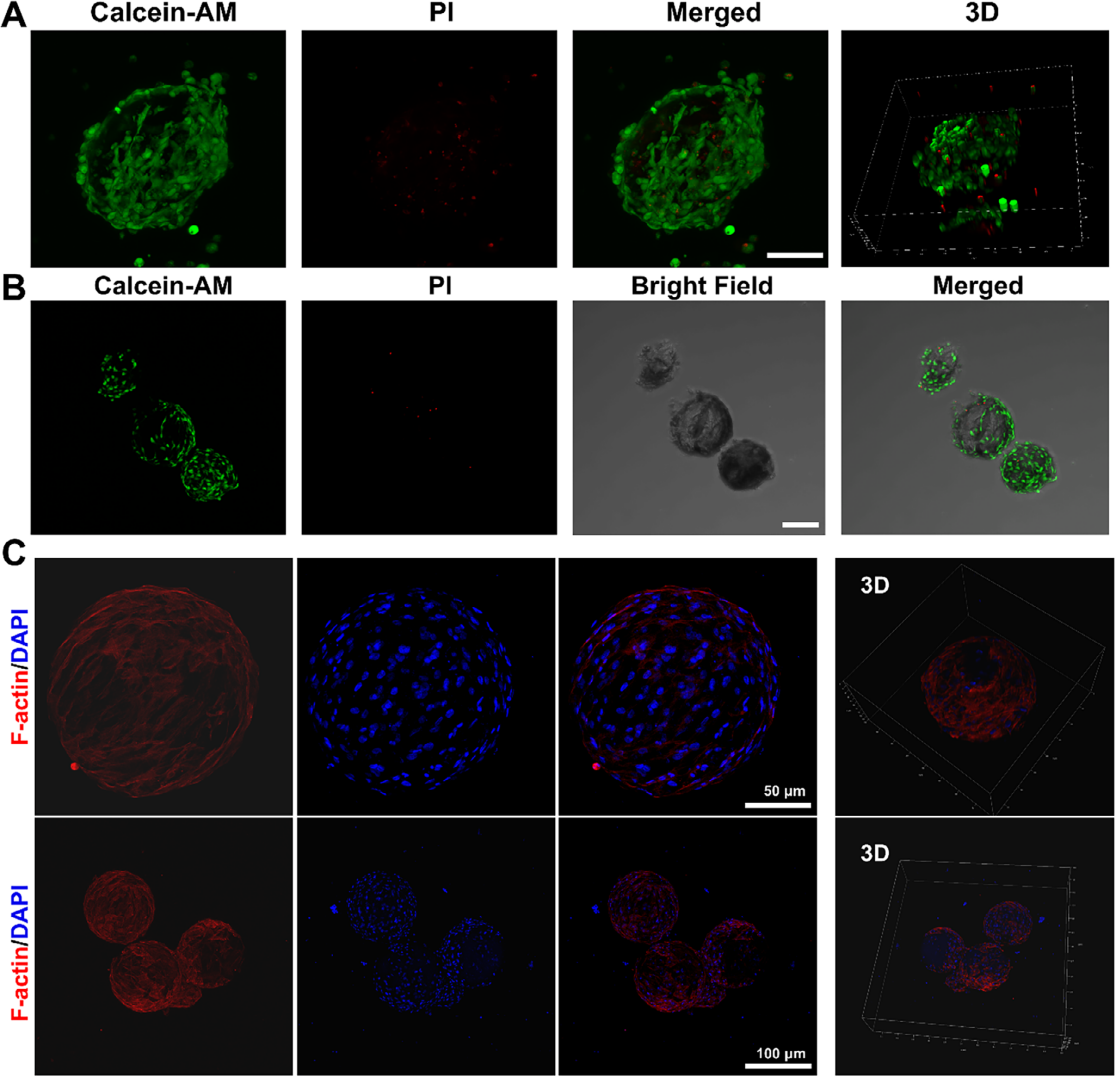
Assessment of chondrocyte viability and stem cell loading capacity of freeze‐dried and non‐freeze‐dried GLPM/Dsp@ChSMA hydrogel microspheres. (A) Calcein‐AM/PI staining images of rat chondrocytes cultured for 48 h with freeze‐dried microspheres (scale bar: 100 µm). (B) Calcein‐AM/PI staining images of rat chondrocytes cultured for 48 h with non‐freeze‐dried microspheres (scale bar: 100 µm). (C) Cytoskeletal staining and 3D reconstruction of rat BMSCs after 48 h of co‐culture with freeze‐dried GLPM/Dsp@ChSMA microspheres.

To assess the potential of GLPM/Dsp@ChSMA microspheres as a 3D scaffold for cartilage repair, we evaluated their compatibility with chondrocytes and BMSCs in 3D co‐culture systems. Calcein‐AM/PI staining of chondrocytes cultured on microspheres revealed strong Calcein‐AM (green) fluorescence with minimal propidium iodide (PI, red) signal, indicating high viability across all time points (Figure 6A,B). Importantly, freeze‐dried microspheres exhibited increased cell adherence and spreading compared to non‐dried counterparts, likely due to enhanced surface roughness and increased porosity introduced during lyophilization.

3D reconstruction via confocal microscopy confirmed homogeneous cell attachment and distribution throughout the microsphere surface, validating the microsphere's ability to support cartilage cell colonization in a physiologically relevant geometry. The hydrated, ECM‐mimicking nature of the ChSMA matrix provides both mechanical and biochemical cues that facilitate cell proliferation and matrix synthesis.

To further investigate stem cell compatibility and cytoskeletal organization, BMSCs were cultured on the microspheres and subjected to actin cytoskeleton staining. As shown in Figure 6C, phalloidin‐labeled F‐actin (red) was well‐organized and extended across the microsphere surface, suggesting active cell spreading and attachment. 4',6‐diamidino‐2‐phenylindole (DAPI) staining of nuclei showed uniform distribution, and 3D image reconstruction revealed dense BMSC coverage across the porous surface. These results indicate that the microspheres provide a mechanically supportive and topographically favorable environment for stem cell integration.

Moreover, BMSCs maintained high viability in both fresh and freeze‐dried microsphere groups, confirming the biocompatibility of the system (Figure S13). The porous microarchitecture not only facilitated nutrient exchange but also enabled dynamic interaction between seeded cells and the hydrogel matrix, which is crucial for successful tissue integration.

Altogether, these findings demonstrate that GLPM/Dsp@ChSMA microspheres offer a highly biocompatible and structurally supportive microenvironment for both chondrocytes and stem cells. Their ability to maintain cell viability, promote cytoskeletal organization, and support 3D colonization highlights their promise as an injectable scaffold for cell‐based cartilage regeneration therapies.

### 2.7 In Vivo Therapeutic Efficacy: Structural and Functional Restoration in OA Model

To validate the therapeutic efficacy of GLPM/Dsp@ChSMA microspheres in vivo, we employed a monosodium iodoacetate (MIA) induced OA rat model followed by weekly IA injections of various microsphere formulations for five weeks. Micro‐computed tomography (micro‐CT) and histological analysis were used to evaluate joint structure restoration, while immunofluorescence and cytokine profiling assessed the local immune response.

**FIGURE 7:**
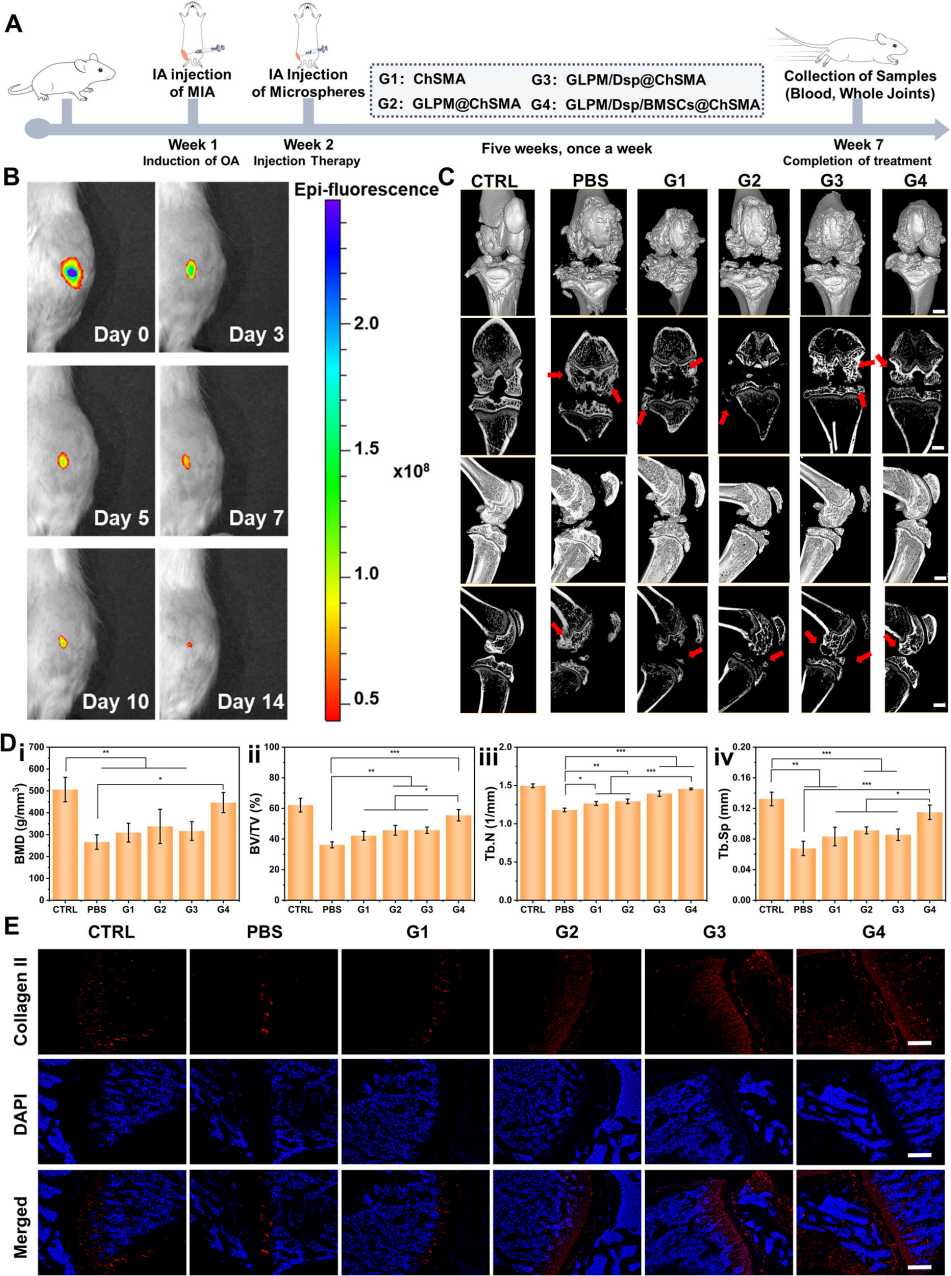
Radiological evaluation of OA treatment outcomes following administration of hydrogel microspheres. (A) Schematic diagram of the in vivo experimental protocol. (B) In vivo degradation behavior analysis of GLPM/Dsp/BMSCs@ChSMA. (C) Micro‐CT images of rat knee joints after 5 weeks of treatment with various microsphere formulations, shown in frontal and lateral views. Red arrows indicate osteophyte regions (scale bar: 2 mm). (D) Micro‐CT‐derived quantitative parameters of bone architecture after 5 weeks of treatment, including (i) BMD, (ii) BV/TV, (iii) Tb.N, and (iv) Tb.Sp (^*^ *p* < 0.05, ^**^ *p* < 0.01, ^***^ *p* < 0.001). (E) Immunofluorescence staining images of type II collagen expression in rat knee cartilage following 5 weeks of treatment with different microspheres (scale bar: 200 µm).

Based on the excellent in vivo clearance of ROS and anti‐inflammatory effects of GLPM/Dsp@ChSMA, further investigation was conducted to explore the therapeutic effects of different groups on OA. Figure 7A illustrates the schematic diagram of microsphere treatment for OA. In week 1, MIA was injected into the joint in situ to construct a rat OA model. In week 2, the rats were divided into groups and treated with IA injections, continuing until week 7. After treatment, the rats were euthanized to collect samples for subsequent analysis. Figure S14 shows the weight changes of the rats in each group over these 7 weeks. The fluctuation in weight gain trend appeared at week 5, which may be due to the slower pathological progression of OA, leading to reduced movement and food intake in the rats after week 5 due to knee pain. In contrast, stable weight gain trends were observed in the CTRL, G3, and G4 groups, suggesting that the pain symptoms of OA in the G3 and G4 groups were lower than those in the PBS group.

To further clarify the in vivo persistence of the microspheres and prevent misinterpretation arising from differences between in vitro and in vivo degradation profiles, we evaluated the actual intra‐articular degradation and metabolic behavior of GLPM/Dsp/BMSCs@ChSMA microspheres using DIR (1,1'‐Dioctadecyl‐3,3,3,3'‐tetramethylindotricarbocyanine Iodide) fluorescence tracing. As shown in Figure 7B, the fluorescence intensity of DIR‐labeled microspheres and encapsulated BMSCs gradually decreased over time following IA injection, reflecting their real‐time degradation and metabolic clearance inside the joint. Notably, by day 14, only faint residual fluorescence remained, indicating that most microspheres had already disassembled and been cleared from the joint cavity. It is important to emphasize that this in vivo degradation behavior represents the actual metabolic fate of microspheres within the complex synovial environment, which differs from the in vitro degradation timeline due to enzyme activity, synovial fluid turnover, mechanical joint motion, and immune‐mediated clearance. Such accelerated clearance in vivo is expected and does not contradict the in vitro degradation results obtained under controlled conditions. Based on this 14‐day intra‐articular residence time, a once‐weekly IA injection regimen was selected to maintain continuous therapeutic availability throughout the treatment period. This dosing frequency ensures that new microspheres are introduced before the previous batch is fully eliminated, thereby sustaining an effective level of antioxidative, anti‐inflammatory, and regenerative activity within the OA joint.

OA clinical symptoms can manifest as subchondral bone sclerosis or cystic lesions leading to osteophyte formation at the joint margins, with significant osteophyte proliferation. Micro‐CT evaluates changes in cartilage calcium deposition and bone density during OA progression by detecting subtle structural alterations in the bone marrow and cortical bone, thereby studying its pathophysiological changes. Subsequently, the therapeutic effects of GLPM/Dsp/BMSCs@ChSMA were investigated through imaging. Micro‐CT scans of the knee joints (Figure 7C) revealed significant osteophyte formation, surface roughness, and subchondral bone sclerosis in the PBS group, consistent with progressive OA. In contrast, joints treated with GLPM/Dsp/BMSCs@ChSMA microspheres (G4) displayed smooth articular surfaces, reduced osteophyte formation, and preserved joint architecture. Quantitative analysis (Figure S15) showed that the relative osteophyte volume in G4 was reduced to ∼20% of that observed in the PBS group. Furthermore, bone volume fraction (BV/TV), one mineral density (BMD), trabecular number (Tb.N), and trabecular separation (Tb.Sp) were significantly improved in G4‐treated joints (Figure 7D), indicating a protective effect against subchondral bone remodeling and structural deterioration. Notably, although OA is frequently associated with subchondral bone sclerosis due to elevated turnover, recent studies have shown that the newly formed bone is often insufficiently mineralized, leading to a paradoxical decrease in BMD. Thus, the reduced BMD observed in the PBS group likely reflects an early‐stage remodeling phase characterized by abnormal bone turnover and defective mineralization, rather than net bone mass loss [54, 55].

Immunofluorescence staining for type II collagen revealed abundant and evenly distributed expression in the G4 group, comparable to healthy controls (Figure 7E). In contrast, type II collagen expression was sparse and disorganized in the PBS group, confirming matrix degradation [56]. This suggests that GLPM/Dsp/BMSCs@ChSMA microspheres effectively restore cartilage‐specific ECM components.

### 2.8 Histological Evaluation of the Therapeutic Effects of Composite Hydrogel Microspheres in OA

Histological staining was conducted to assess the protective and regenerative effects of GLPM and composite hydrogel microspheres on cartilage in vivo. Safranin O‐Fast Green and hematoxylin and eosin (H&E) staining revealed that in the control group, cartilage surfaces remained smooth and structurally intact. In contrast, the PBS group exhibited pronounced osteoarthritic features, including cartilage thinning, surface roughness, fissures, ulcerations, and significant loss of proteoglycans and collagen. H&E staining further confirmed severe tissue degeneration, with rough surfaces, vertical fissures, matrix erosion, cellular degeneration, and disorganized chondrocyte arrangement. Notably, cartilage in the groups treated with 5 and 50 µg/mL GLPM showed smoother surfaces, clearer tissue architecture, and better‐preserved boundaries compared to the PBS group, although signs of matrix degradation were still evident and full regenerative effects were not achieved (Figure S16). These findings suggest that higher GLPM concentrations mitigate cartilage damage but highlight the need for additional strategies to fully restore cartilage integrity in OA.

**FIGURE 8:**
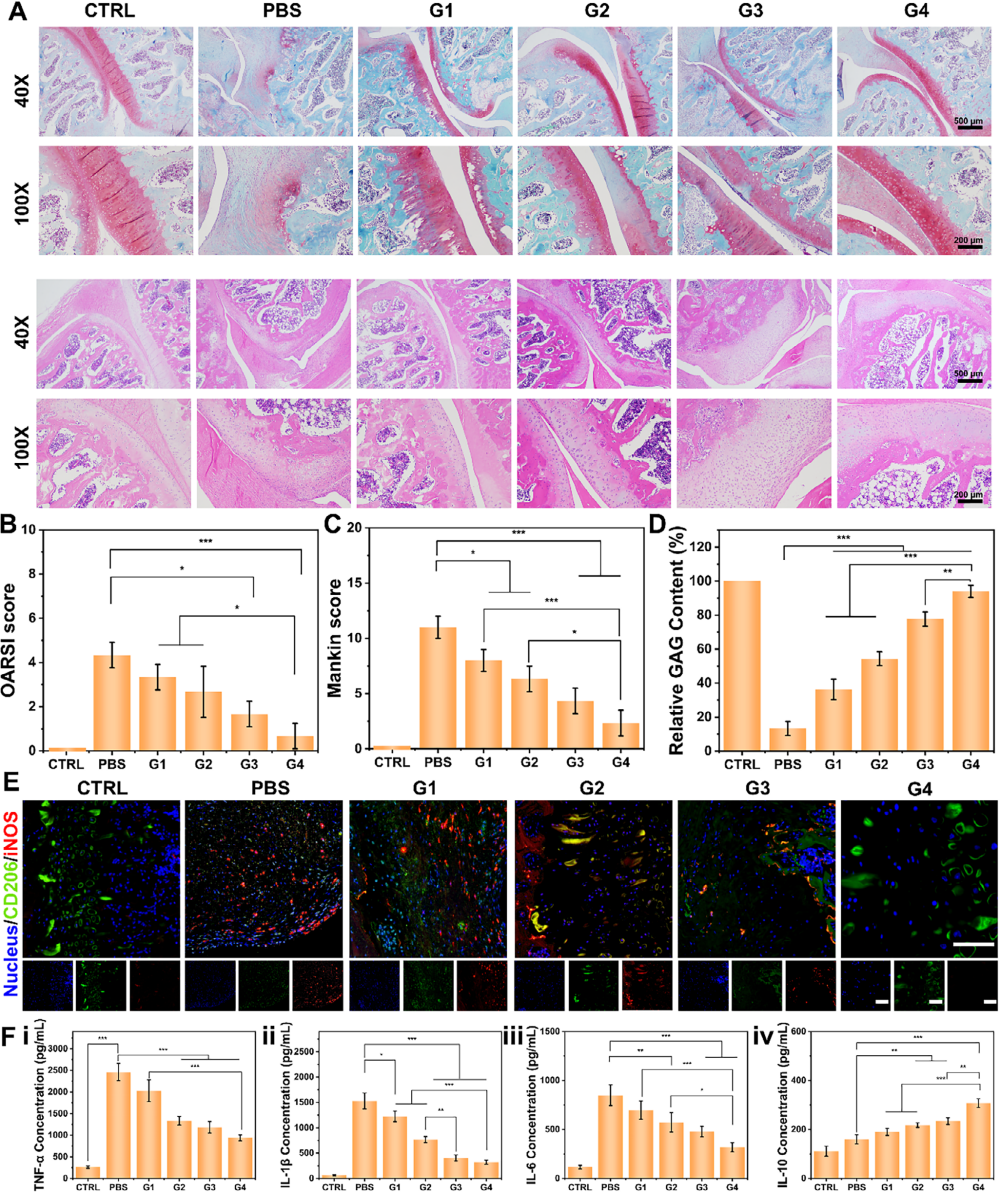
Histological and anti‐inflammatory evaluation of OA treatment following administration of hydrogel microspheres. (A) Representative images of cartilage sections stained with Safranin O‐Fast Green and H&E after 5 weeks of treatment with different microsphere formulations. (B) Quantitative OARSI scores for cartilage degeneration across treatment groups. (C) Mankin scores for cartilage histopathological assessment. (D) Relative GAG content in cartilage tissue. (E) Immunofluorescence staining images of knee joint sections showing inflammatory markers after 5 weeks of treatment with various microspheres (scale bar: 50 µm). (F) Analysis of inflammatory cytokine levels in rat serum following 5 weeks of treatment, showing the effects of different microspheres on (i) suppression of pro‐inflammatory cytokines TNF‐α, IL‐1β, and IL‐6, and (iv) upregulation of the anti‐inflammatory cytokine IL‐10 (*n* = 6; ^*^ *p* < 0.05, ^**^ *p* < 0.01, ^***^ *p* < 0.001).

Further investigation focused on G4 treatment, representing the composite GLPM/Dsp/BMSCs@ChSMA microspheres. Safranin O‐Fast Green staining demonstrated severe cartilage erosion and proteoglycan loss in the PBS group, whereas joints treated with G4 showed continuous cartilage surfaces with restored proteoglycan content and well‐organized chondrocyte columns (Figure 8A). H&E staining of the G4 group confirmed preservation of cartilage zonation and minimal matrix disruption [57]. Histological scoring using Osteoarthritis Research Society International (OARSI) and Mankin criteria revealed significantly lower scores in the G4 group compared to PBS, indicating superior cartilage preservation and regenerative efficacy (Figure 8B,C) [58, 59]. Specifically, OARSI and Mankin scores in the G4 group decreased by 88% and 78%, respectively, reflecting minimal surface erosion and preserved cartilage architecture. Safranin O staining confirmed near‐normal glycosaminoglycan (GAG) content, supporting the potent chondroprotective effect of GLPM/Dsp/BMSCs@ChSMA microspheres.

Semi‐quantitative analysis using ImageJ software demonstrated that GAG content in the G4 group recovered to levels comparable to the control group. These findings suggest that G4 treatment nearly reversed OA‐associated proteoglycan loss by promoting GAG synthesis and inhibiting matrix degradation, with the structural repair closely resembling healthy cartilage architecture (Figure 8D). Such results highlight the potential of GLPM/Dsp/BMSCs@ChSMA microspheres as an effective strategy for OA therapy.

To explore the underlying mechanisms, immunofluorescence staining was conducted to examine macrophage polarization within the synovial tissue. In the PBS group, strong iNOS expression indicated a predominance of pro‐inflammatory M1 macrophages. However, G4‐treated joints displayed reduced iNOS levels and increased expression of CD206, suggesting a shift toward an anti‐inflammatory M2 macrophage phenotype (Figure 8E). The MnO_2_ nanozymes, by reducing oxidative stress, may suppress NF‐κB/MAPK pathways that otherwise inhibit M2 polarization under inflammatory conditions [60, 61, 62]. Meanwhile, BMSCs are known to exert paracrine effects by releasing IL‐10, TGF‐β, and PGE2, which further facilitate M2 reprogramming [63, 64, 65]. This transition likely results from the combined ROS scavenging properties of MnO_2_ and the anti‐inflammatory effects of dexamethasone.

ELISA analysis further supported these observations. Compared to PBS and other controls, the G4 group exhibited significantly lower levels of pro‐inflammatory cytokines TNF‐α, IL‐6, and IL‐1β, along with elevated levels of the anti‐inflammatory cytokine IL‐10 (Figure 8F; Figure S17). In combination, these data suggest that the composite microspheres regulate the immune microenvironment through both redox modulation and paracrine immune crosstalk. These results indicate that the composite microspheres effectively modulate the inflammatory microenvironment, promoting tissue repair and suppressing cartilage degeneration.

To ensure biosafety, major organs from treated rats were collected and examined via H&E staining, alongside hematological analyses. Both PBS and GLPM‐treated groups displayed intact tissue architecture and normal cellular morphology, with no significant alterations in blood parameters such as hemoglobin concentration, mean corpuscular volume, or red cell distribution width (Figures S18 and S19). These findings confirm that the developed materials exhibit low systemic toxicity and are safe for potential therapeutic applications in OA.

Collectively, these in vivo data demonstrate that GLPM/Dsp/BMSCs@ChSMA microspheres, particularly when incorporating stem cells, offer multifaceted benefits for OA treatment. They effectively modulate inflammation, reduce oxidative stress, preserve cartilage structure, and contribute to functional joint restoration, positioning this biomaterial system as a promising approach for localized and minimally invasive OA therapy.

## 3 Conclusions

In this study, we developed a versatile, clinically relevant GLPM/Dsp@ChSMA hydrogel microsphere platform for the multimodal treatment of OA, integrating ROS scavenging, anti‐inflammatory drug delivery, and stem cell‐enabled cartilage regeneration within a single injectable construct. The microspheres were fabricated via a UV‐assisted microfluidic technique, incorporating redox‐active manganese nanozymes, Dsp, and a photocrosslinkable ChSMA matrix.

The synthesized GLPM nanoparticles exhibited robust ROS‐scavenging capacity and a well‐defined Mn (IV) oxidation state, enabling targeted oxidative microenvironment modulation. ChSMA‐based microgels provided a highly porous, ECM‐mimetic network with tunable injectability and mechanical compliance. The resulting microspheres demonstrated long‐term drug release, inflammation‐responsive degradation, and adaptive swelling behavior, aligning with the dynamic physiological cues of the IA cavity.

Functionally, the microspheres attenuated ROS‐mediated cytotoxicity and reprogrammed macrophages toward a pro‐regenerative M2 phenotype, while supporting the co‐culture of chondrocytes and BMSCs with enhanced cellular adhesion, cytoskeletal organization, and 3D integration. In vivo, IA delivery of GLPM/Dsp@ChSMA microspheres significantly mitigated OA progression in a rat model, as evidenced by cartilage preservation on micro‐CT, enhanced COL II expression, reduced pro‐inflammatory cytokine production, and a marked increase in M2 macrophage infiltration. Strikingly, the incorporation of BMSCs further amplified regenerative outcomes, reflecting the system's potential for cell‐assisted tissue repair in challenging joint environments.

Overall, this work presents a modular and translationally promising microsphere system that co‐targets inflammation, oxidative stress, and extracellular matrix degeneration in OA. Through its injectable format, biomechanical adaptability, and immune‐modulatory functionality, this platform offers a minimally invasive, multi‐targeted strategy for localized OA therapy, while laying the groundwork for next‐generation hydrogel systems capable of precision cell and drug delivery in chronic musculoskeletal disorders. Nevertheless, we acknowledge that the MIA‐induced rat model used here only partially recapitulates the complexity of human OA. Species differences in cartilage biology, inflammatory progression, and subchondral bone remodeling may influence therapeutic responses. Future studies will incorporate multiple OA models, including genetically engineered and mechanically induced models, to better capture human disease heterogeneity and to strengthen the translational relevance of this microsphere platform.

## 4 Materials and Methods

### 4.1 Materials

Ultra‐pure water was obtained from a Direct‐Q 5UV system (Millipore, China). Ganoderma lucidum spore powder was purchased from Xi'an Xiaosheng Biotechnology. Anhydrous ethanol, trichloromethane, n‐hexane, sodium periodate (≥99.9%), hydrogen peroxide, sodium iodoacetate, glacial acetic acid, sodium acetate, potassium bromide, carbonyldiimidazole, and Span‐80 were supplied by China National Pharmaceutical Group. Dimethyl sulfoxide (≥99%) and lithium phenyl‐2,4,6‐trimethylbenzoylphosphinate (LAP, 99%) were from Macklin (Shanghai, China). Sodium alginate (≥97%) and TMB were from Sigma–Aldrich (USA). N‐hydroxysuccinimide (≥96%) and EDC (≥98%) were from Aladdin (Shanghai, China). Cy5.5 dye was purchased from Xi'an Deermeta Biotechnology. Collagenase II (≥125 CDU/mg) was from Solarbio (Beijing, China). Dexamethasone sodium phosphate was from Qilu Pharmaceutical. DMEM, fetal bovine serum (FBS), penicillin‐streptomycin, and trypsin ethylenediaminetetraacetic acid (EDTA) were obtained from Biological Industries (Israel). Specialized media for rat chondrocytes and RAW264.7 macrophages were from Procell (Wuhan, China). CCK‐8, Calcein‐AM/PI staining kits, ROS detection kits (DCFH‐DA), and ELISA kits for IL‐6, IL‐1β, IL‐10, and TNF‐α were purchased from Solarbio (Beijing, China) and Xinyu Sheng Biotechnology (China). DIR was from Meilunbio (Guangzhou, China). Primary antibodies against CD68, iNOS, CD206, collagen II, and corresponding secondary antibodies were from Abcam (UK) and Solarbio. All other reagents were of analytical or cell culture grade.

### 4.2 Synthesis and Characterization of GLPM

To synthesize GLPM, a 20 mg/mL GLP solution and a 0.01 m NaMnO_4_·H_2_O aqueous solution were first prepared. GLP was diluted to 10 different concentrations (0.1–1.0 mg/mL) and mixed with equal volumes of the NaMnO_4_ solution in Eppendorf tubes (1 mL each). The mixtures were stirred for 24 h at room temperature, and the reaction progression was monitored using UV–vis spectroscopy. Based on absorbance profiles, the optimal ratio was selected, and the resulting suspensions were centrifuged to collect the precipitates, which were lyophilized to obtain GLPM powders.

FTIR spectroscopy was used to analyze functional group characteristics. GLP, pristine MnO_2_, and GLPM powders were mixed with dried KBr, pressed into pellets, and scanned in the range of 4000–400 cm^−1^. XRD patterns were acquired using a Cu‐Kα source (λ = 1.5406 Å) at 40 kV and 30 mA with a scan rate of 10°/min over a 5–90° range. For morphological examination, dried GLPM samples were sputter‐coated with gold and analyzed by SEM. TEM was performed by depositing 10 µL of diluted GLPM dispersion onto copper grids, followed by natural drying. DLS was used to assess particle size distribution using a Zetasizer Nano ZS instrument (Malvern, UK).

### 4.3 Evaluation of Antioxidant Properties

The antioxidant potential of GLPM nanoparticles was comprehensively assessed by evaluating their ability to neutralize various free radicals, including OH, DPPH radicals, and ABTS^+^· cations, using a series of colorimetric assays.

#### 4.3.1 OH Scavenging Assay

To generate hydroxyl radicals via a Fenton‐type reaction, 1 mL of H_2_O_2_ (50 mm), 1 mL of FeSO_4_ (5 mm), and 1 mL of 0.2 m sodium acetate‐acetic acid buffer (pH 4.5) were mixed thoroughly. GLPM nanoparticle dispersions at varying concentrations (0, 25, 50, 75, 100, 150, 200 µg/mL) were added (500 µL each) and incubated at 37 °C for 1 h. Subsequently, 500 µL of 10 mm TMB solution was introduced, and the mixture was gently shaken for 10 min. The absorbance spectra from 500 to 750 nm were recorded using a UV–vis spectrophotometer, with the decrease in absorbance at 652 nm indicating *·*OH scavenging activity. Each test was performed in triplicate (*n* = 3).

#### 4.3.2 DPPH·Radical Scavenging

GLPM dispersions (0–200 µg/mL) were mixed in a 1:1 ratio with 1 mL of freshly prepared 1 mm DPPH ethanol solution. The mixtures were incubated in the dark at 37 °C for 30 min. After centrifugation (10,000 rpm, 10 min), the supernatants were collected, and UV–vis spectra were acquired over the 400–800 nm range. A reduction in absorbance at ∼517 nm was used as an indicator of DPPH radical scavenging.

#### 4.3.3 ABTS^+^·Cation Radical Scavenging

The ABTS^+^· radical cation solution was prepared by reacting 30 mg ABTS with 30 mg potassium persulfate in 100 mL deionized water, followed by a 12 h incubation in the dark. After dilution to an optical density of 0.70 ± 0.02 at 734 nm, 1 mL of the ABTS^+^· solution was mixed with 1 mL of GLPM dispersions at varying concentrations (0–200 µg/mL) and incubated at 37 °C for 30 min in the dark. The decrease in absorbance at 734 nm was used to quantify the ABTS^+^· radical scavenging capacity.

### 4.4 Synthesis of ChSMA

ChSMA was synthesized by chemically modifying the hydroxyl and amine groups of ChS with methacrylic anhydride (MAA), following a slightly adapted protocol from previous reports [66]. Briefly, 2.0 g of ChS was dissolved in 50 mL of PBS under magnetic stirring. Subsequently, 16.9 mL of MAA was added dropwise while the solution pH was maintained at 8.0 using 5 N NaOH. The reaction was allowed to proceed at room temperature for 2 h, followed by continued stirring at 4 °C for 24 h to ensure complete methacrylation. After the reaction, the mixture was subjected to ethanol precipitation to remove unreacted reagents. The precipitated product was collected by centrifugation and dissolved in deionized water, followed by dialysis (MWCO: 3.5 kDa) against deionized water for 3–5 days. The dialyzed solution was subsequently lyophilized to obtain ChSMA as a white sponge‐like solid. To confirm successful methacrylation, both native ChS and ChSMA samples (5 mg each) were dissolved in D_2_O and analyzed by ^1^H NMR (Bruker, 400 MHz). The degree of methacrylation was estimated by integrating the peaks corresponding to the vinyl protons (δ = 5.5–6.5 ppm) of the methacrylate groups relative to the proton signals from the sugar backbone.

### 4.5 Microfluidic Fabrication and Structural Characterization of Hydrogel Microspheres GLPM/Dsp@ChSMA

Hydrogel microspheres were fabricated using a UV‐assisted microfluidic emulsion approach to ensure high monodispersity and precise control over microsphere morphology [67]. The aqueous phase consisted of PBS containing 10 wt.% ChSMA, 200 µg/mL GLPM nanoparticles, and 0.25 wt.% LAP as the photoinitiator. This precursor solution was loaded into a 1 mL syringe fitted with a 28 G needle and injected as the dispersed phase. The continuous oil phase was prepared from a mixture of paraffin oil (85 wt.%) and Span 80 (15 wt.%) to stabilize the forming droplets. Both aqueous and oil phases were introduced into a capillary microfluidic device at controlled flow rates of 5 and 500 µL/min, respectively. The shear force at the flow‐focusing junction generated uniform aqueous droplets, which were immediately photo‐crosslinked under 365 nm UV light (10 mW/cm^2^, 10 s) to form stable hydrogel microspheres. The resulting particles were collected and sequentially washed with isopropanol and PBS to remove oil residues and surfactants. Purified microspheres were stored in PBS at 4 °C for further analysis.

Microsphere morphology was evaluated using optical microscopy (Olympus IX71), and size distribution was quantified via ImageJ based on over 100 randomly selected particles.

To characterize the surface morphology and elemental composition of the microspheres, SEM and EDS were employed. Microspheres were first flash‐frozen in liquid nitrogen and lyophilized to maintain structural features. The dried samples were mounted on conductive carbon adhesive tape and sputter‐coated with a thin layer of gold to enhance conductivity. SEM imaging was performed using a field‐emission scanning electron microscope (FEI Nova NanoSEM) under high‐vacuum mode. For elemental analysis, EDS mapping was conducted on GLPM/Dsp@ChSMA microspheres using the same instrument equipped with an EDS detector. Mapping was carried out under consistent operating parameters to identify and visualize the spatial distribution of key elements, including Mn, P, S, and O. All elemental signals were recorded and analyzed using built‐in software to assess component dispersion across the microsphere matrix.

### 4.6 Mechanical and Tribological Evaluation of Hydrogel Microspheres

Inspired by previous studies on the biomechanical adaptation and lubrication of IA microspheres, we systematically evaluated the mechanical and tribological performance of ChSMA and GLPM@ChSMA hydrogel microspheres [29, 48].

To assess the microspheres' mechanical resilience, single‐particle compression tests were performed in PBS using a precision micromechanical testing platform. A custom‐built stainless‐steel flat‐ended probe (diameter = 0.56 mm) was used to apply vertical compressive force on individual microspheres. Each microsphere was submerged in PBS and compressed from 100% to 75% of its original height. Real‐time force and displacement data were captured using a high‐sensitivity load cell. The resulting force‐time, displacement‐time, and force‐displacement curves were analyzed to characterize microsphere deformation behavior.

Inspired by previous studies on the biomechanical adaptation and lubrication of IA microspheres, we systematically evaluated the mechanical and tribological performance of ChSMA and GLPM@ChSMA hydrogel microspheres. Frictional behavior was assessed in reciprocating mode with a stroke amplitude of 4 mm and a sliding frequency of 1 Hz for 15 min per test. A typical steel‐PTFE tribo‐pair was employed, comprising a highly polished stainless‐steel spherical counterpart and a PTFE plate with a 5 mm contact diameter. Each hydrogel formulation was dispersed in phosphate‐buffered saline (PBS) at a concentration of 5 mg/mL. PBS alone served as the negative control to rule out nonspecific interference. During testing, COF values were continuously recorded as a function of time. All experiments were performed in triplicate to ensure reproducibility.

### 4.7 Degradation and Drug Release Behavior

To assess the biodegradability of the hydrogel microspheres under pathophysiologically relevant conditions, GLPM/Dsp@ChSMA microspheres were incubated in various degradation media. Specifically, the microspheres were immersed in PBS containing 2 U/mL of collagenase type II (CII), simulating enzymatic degradation in the joint space. Additionally, an inflammatory‐mimicking buffer composed of PBS supplemented with 100 µm H_2_O_2_ was used to simulate oxidative stress conditions. PBS of pH 6.5 served as the control. All samples were incubated in a shaker incubator at 37 °C and 100 rpm. The medium was refreshed every 48 h. At predetermined time points (days 1, 3, 5, 7, 14, 21, and 28), the remaining microspheres were collected, lyophilized, and weighed to determine the residual mass. Degradation was expressed as the percentage of initial dry weight remaining. Each condition was tested in triplicate. To evaluate the in vitro drug release profile, microspheres were incubated in PBS containing 2 U/mL CII at 37 °C. At regular intervals (every 2 days), 3 mL of supernatant was withdrawn and replaced with an equal volume of fresh buffer. The cumulative release of Dsp was quantified by measuring the absorbance at 240 nm using a UV–vis spectrophotometer. All samples were stored at −20 °C prior to analysis. In addition, the swelling behavior of the microspheres was assessed by incubating them in PBS at 37 °C and collecting weight measurements at multiple time points (10, 20, 30, 40, 50 min, and 1, 2, 3, 6 h). The degree of swelling was calculated as the percentage increase in mass relative to the dry weight. Each experiment was performed in triplicate.

### 4.8 Evaluation of Microsphere Biocompatibility

To assess the cytocompatibility and biological effects of the hydrogel microspheres, extract media were prepared by incubating different formulations of microspheres in complete culture medium. Specifically, ChSMA, Dsp@ChSMA, GLP/Dsp@ChSMA, and GLPM/Dsp@ChSMA microspheres were first sterilized by immersion in 75% ethanol for 30 min, followed by thorough rinsing with sterile PBS three times to remove residual ethanol. Each microsphere type was then added to DMEM at a concentration of 2% w/v and incubated in a shaker incubator at 200 rpm and 37 °C for 24 h. The supernatants, defined as microsphere extracts, were collected by centrifugation (1000 rpm, 5 min) and filtered through 0.22 µm syringe filters to ensure sterility.

The biocompatibility of GLPM nanoparticles was comprehensively assessed using rat chondrocytes via cell viability assays, Calcein‐AM/PI staining, and hemolysis testing.

Rat primary chondrocytes were seeded into 24‐well plates at a density of 1 × 10^4^ cells per well and incubated for 24 h at 37 °C with 5% CO_2_. After adherence, cells were treated with GLPM dispersed in DMEM at varying concentrations (0.5, 1, 5, 10, and 50 µg/mL) and further incubated for 24 h. Subsequently, 100 µL of a 1:10 diluted CCK‐8 working solution was added to each well, and plates were incubated for an additional 2.5 h. Absorbance was measured at 450 nm using a microplate reader (BioTek), and cell viability was calculated relative to untreated controls.

(1) $$\mathrm{Cell}\;\mathrm{viability}\;(\%)=\frac{OD_{\mathrm{test}}-OD_{\mathrm{blank}}}{OD_{\mathrm{control}}-OD_{\mathrm{blank}}}\times 100\%\;\;$$

where the OD_test_, OD_blank_, and OD_control_ represent the absorbance values of the test, blank, and control groups at 450 nm, respectively.

To evaluate the membrane integrity and viability of chondrocytes exposed to GLPM, cells were seeded in 24‐well plates at 1 × 10^5^ cells per well and cultured overnight. The following day, cells were treated with GLPM suspensions at concentrations identical to those used in the CCK‐8 assay and incubated for 1 or 2 days. After incubation, cells were gently rinsed with PBS and stained with a 1:1000 diluted Calcein‐AM/PI solution for 30 min at 37 °C in the dark. Fluorescence images were acquired using an inverted fluorescence microscope (Olympus IX71).

Fresh rat blood was collected via orbital puncture and anticoagulated. Red blood cells (RBCs) were isolated by centrifugation at 2000 rpm for 5 min and washed repeatedly with PBS until the supernatant was clear. GLPM was dispersed in PBS at five concentrations (0.5, 1, 5, 10, and 50 µg/mL). Each dispersion (1 mL) was mixed with 1 mL of a 2% v/v RBC suspension in PBS and incubated at 37 °C for 2 h. PBS and distilled water served as negative (blank) and positive controls, respectively. After incubation, suspensions were centrifuged, and 100 µL of the supernatant from each sample was transferred to a 96‐well plate for absorbance measurement at 540 nm.

The hemolysis rate (%) was calculated using the following formula:

(2) $$\mathrm{Hemolysis}\;\left(\%\right)=\frac{OD_{\mathrm{Experiment}\;\mathrm{group}}-OD_{\mathrm{Blank}\;\mathrm{group}}}{\left(OD_{\mathrm{Positive}\;\mathrm{group}}-OD_{\mathrm{Blank}\;\mathrm{group}}\right)}\times 100\%$$

where the OD_Experiment group_, OD_Blank group_, and OD_Positive group_ represent the absorbance values of the sample, PBS, and deionized water at 540 nm, respectively.

### 4.9 Chondrogenic Differentiation of BMSCs on Microspheres

To evaluate the chondrogenic potential of the microspheres, BMSCs were seeded onto GLPM@ChSMA or ChSMA microspheres. Briefly, freeze‐dried microspheres were sterilized under UV for 30 min, pre‐wetted in complete medium, and dispersed in 24‐well plates. Passage 3 BMSCs were added at a density of 2 × 10^5^ cells/well and allowed to attach for 12 h in standard culture medium. Thereafter, the medium was replaced with chondrogenic differentiation medium composed of high‐glucose DMEM supplemented with 1% insulin–transferrin–selenium, 50 µg/mL ascorbic acid, 100 nm dexamethasone, 1 mm sodium pyruvate, 1% penicillin/streptomycin, and 10 ng/mL TGF‐β3. Cultures were maintained at 37°C in a humidified 5% CO_2_ atmosphere for up to 14 days, and the medium was refreshed every 2–3 days. At predetermined time points (days 3, 7, and 14), samples were washed with PBS, fixed with 4% paraformaldehyde for 20 min, and stained with 1% Alcian blue solution (pH 1.0) for 30 min to visualize sGAG deposition. Excess dye was removed by thorough washing with 0.1 N HCl followed by PBS, and the samples were imaged under a light microscope.

### 4.10 In Vitro Immunomodulatory Evaluation

To investigate the immunoregulatory effects of the hydrogel microspheres under inflammatory conditions, murine RAW 264.7 macrophages were employed to assess phenotypic polarization and cytokine responses. Cells in logarithmic growth phase were harvested and seeded into confocal‐compatible 24‐well plates at a density of 1 × 10^5^ cells/well. After overnight incubation under standard culture conditions (37 °C, 5% CO_2_), macrophages, excluding the negative control group, were primed with LPS (100 ng/mL) for 24 h to induce pro‐inflammatory (M1) activation. Following LPS stimulation, the cells were treated for an additional 24 h with 50 µg/mL extract media derived from either GLPM nanoparticles or GLPM/Dsp@ChSMA hydrogel microspheres. After treatment, culture supernatants were collected by centrifugation (2500 rpm, 30 min, 4 °C) for subsequent cytokine analysis. Adherent cells were washed three times with PBS and fixed with 4% paraformaldehyde for 15 min at room temperature. Subsequent immunofluorescence staining was performed to assess macrophage phenotype. Fixed cells were permeabilized with 0.2% Triton X‐100 for 10 min and blocked with 5% normal goat serum for 30 min. Cells were incubated overnight at 4 °C with dual primary antibodies targeting CD68 (pan‐macrophage marker, mouse monoclonal) and either inducible nitric oxide synthase (iNOS, M1 marker, rabbit polyclonal) or CD206 (M2 marker, rabbit polyclonal), each diluted 1:100 in blocking buffer. The following day, cells were washed and incubated with fluorescently labeled secondary antibodies: DyLight 488‐conjugated goat anti‐mouse IgG (green) and DyLight 649‐conjugated goat anti‐rabbit IgG (red), both diluted 1:500. Nuclear counterstaining was performed using DAPI for 10 min. After final PBS washes, stained samples were mounted with antifade reagent and imaged using CLSM (Leica SP8). Appropriate laser lines and filter sets were employed to distinguish CD68^+^/iNOS^+^ and CD68^+^/CD206^+^ double‐positive cells, allowing quantitative assessment of macrophage polarization.

### 4.11 3D Cell Encapsulation and Viability Assessment in Hydrogel Microspheres

To assess the cytocompatibility and cell‐encapsulation potential of the GLPM/Dsp@ChSMA hydrogel microspheres, 3D co‐culture experiments were performed using primary rat chondrocytes and BMSCs. Both cell types were seeded at a density of 4 × 10^6^ cells / 50 mL centrifuge tube, and incubated with either freeze‐dried or freshly prepared, UV‐sterilized microspheres (5 mg/tube) in serum‐free medium. Tubes were sealed and maintained under continuous agitation in a shaker incubator at 37 °C and 150 rpm for 48 h to facilitate cell‐microsphere interaction and adhesion.

After incubation, the microspheres were gently collected and transferred to confocal‐compatible culture plates. For live/dead assessment, samples were stained using Calcein‐AM (green, live cells) and PI (red, dead cells) diluted at 1:1000 in PBS. After 30 min of incubation at 37 °C in the dark, samples were washed with PBS and imaged using CLSM. Z‐stack images were acquired and reconstructed to visualize 3D cell viability distribution within the microspheres.

To further confirm the cytoskeletal organization of cells encapsulated within the microspheres, F‐actin and nuclear staining were performed. Microspheres containing BMSCs were fixed in 4% paraformaldehyde for 15 min, permeabilized with 0.1% Triton X‐100, and blocked with 1% BSA in PBS. Actin filaments were stained using Actin‐Tracker Red (1:100 dilution), followed by DAPI counterstaining. Samples were imaged under CLSM to reveal cell spreading, cytoskeletal integrity, and nuclear morphology within the 3D hydrogel network.

### 4.12 Establishment of OA Animal Model

An experimental OA model was established in male Sprague‐Dawley (SD) rats via IA injection of MIA, a widely used chemical agent that induces cartilage degeneration resembling human OA pathology [68]. All animal‐related experiments were approved and executed under the guidelines approved by the Institutional Animal Care and Use Committee of Huaqiao University (No. A2024015) and following the guidelines from the Administration of Affairs Concerning the Experimental Animals of China.

Male SD rats (8 weeks old, weighing 220–250 g) were anesthetized using isoflurane inhalation delivered via a small‐animal gas anesthesia system (induction at 3–4%, maintenance at 1.5–2%). Once anesthesia was confirmed, rats were placed in the supine position, and hair around the right knee joint was removed. The knee was flexed at approximately 90°. Using aseptic technique, 50 µL of 50 mg/mL MIA solution was injected into the IA space of the right knee using a 30 G needle. Hemostasis was achieved with a sterile cotton swab post‐injection.

DIR dye was used to monitor the in vivo degradation and metabolic clearance of microspheres. GLPM/Dsp/BMSCs@ChSMA microspheres were incubated with DIR solution (5 µg/mL, 30 min), followed by repeated washing to remove unbound dye. DIR‐labeled microspheres (50 µL) were injected into the rat knee joint under isoflurane anesthesia. Fluorescence imaging was performed at Day 0, 3, 5, 7, 10, and 14 using an IVIS Spectrum system (Ex 748 nm, Em 780–820 nm). Fluorescence signal within a fixed ROI over the joint was quantified to evaluate the temporal loss of signal intensity. The progressive decline in fluorescence reflects actual intra‐articular degradation, synovial clearance, and metabolic elimination of the microspheres under dynamic physiological conditions, which may differ from in vitro degradation behavior.

Seven days after model induction, OA rats were randomly assigned into five groups (*n* = 6): PBS group (negative control), ChSMA group (G1), GLPM@ChSMA group (G2), GLPM/Dsp@ChSMA group (G3), and GLPM/Dsp/BMSCs@ChSMA group (G4). The healthy contralateral limbs served as non‐OA controls. IA injections were performed weekly under anesthesia for a total of five weeks. Rats in the control and PBS groups received 50 µL of sterile PBS, whereas the treatment groups received 50 µL of microsphere suspension containing equivalent dosages of respective formulations.

At the end of the treatment period, animals were sacrificed, and knee joints were harvested for micro‐CT scanning, histological evaluation, and molecular analysis.

### 4.13 Micro‐Computed Tomography and Histological Assessments

To evaluate the structural and therapeutic outcomes of the IA microsphere treatments in vivo, micro‐CT and histological analyses were performed on harvested rat knee joints at the end of the 5‐week intervention period.

Following euthanasia, the right knee joints were excised and fixed in 4% paraformaldehyde for 48 h. Specimens were then subjected to high‐resolution micro‐CT scanning (Bruker SkyScan 1176, Belgium) at a voxel size of 20 µm to assess subchondral bone remodeling and osteoarthritic changes. Quantitative parameters including BMD, BV/TV, Tb.N, Tb.Sp, and relative osteophyte volume were analyzed using CTAn software (Bruker, Belgium).

After scanning, samples were washed with PBS and decalcified in 10% EDTA solution (pH 7.4) for 3–4 weeks at 4 °C with regular solution changes every 3 days until tissues became pliable. Decalcified joints were dehydrated, paraffin‐embedded, and sectioned at 5 µm thickness.

Histological assessment was conducted using H&E staining to examine overall joint architecture and inflammatory infiltration, and Safranin O‐Fast Green staining to assess cartilage proteoglycan content and cartilage degradation severity. Histological scoring was performed according to the OARSI guidelines by two independent blinded observers. Representative sections from each group were compared to assess therapeutic efficacy in terms of cartilage preservation and osteoarthritic lesion mitigation.

### 4.14 Immunohistochemical and Immunofluorescent Staining

To further evaluate cartilage matrix preservation and local immune responses within the joint microenvironment, immunohistochemical (IHC) and immunofluorescent (IF) staining were performed on paraffin‐embedded sections obtained from the OA rat knee joints.

#### 4.14.1 Type II Collagen Immunohistochemistry

Sections were deparaffinized in xylene, rehydrated through graded ethanol, and subjected to antigen retrieval by heating in citrate buffer (pH 6.0) using a microwave (medium‐high for 8 min, pause for 8 min, then medium‐low for 7 min). After natural cooling, slides were rinsed in PBS, and endogenous peroxidase activity was quenched. Non‐specific sites were blocked using 5% goat serum for 1 h at room temperature. Subsequently, sections were incubated overnight at 4 °C with rabbit anti‐collagen II primary antibody (1:100). After washing, sections were incubated with DyLight 649‐conjugated goat anti‐rabbit IgG secondary antibody (1:200) for 1 h at room temperature in the dark. Nuclear counterstaining was performed with DAPI. Fluorescence images were acquired using a CLSM, and signal intensity was quantified using ImageJ.

#### 4.14.2 Macrophage Phenotype Immunofluorescence

Adjacent sections were similarly processed and blocked, followed by overnight incubation with dual primary antibodies: mouse anti‐CD68 (pan‐macrophage marker, 1:100) combined with either rabbit anti‐iNOS (M1 phenotype, 1:100) or rabbit anti‐CD206 (M2 phenotype, 1:100). After PBS washes, slides were incubated for 1 h at room temperature with DyLight 488‐conjugated goat anti‐mouse IgG (green) and DyLight 649‐conjugated goat anti‐rabbit IgG (red), each diluted 1:500. Nuclei were stained with DAPI (10 µg/mL, 5–10 min), and sections were mounted using antifade reagent.

CLSM was used to visualize and record fluorescent signals, and excitation/emission channels were selected to differentiate co‐localized CD68^+^/iNOS^+^ and CD68^+^/CD206^+^ populations. Quantitative analysis of M1/M2 macrophage ratios was performed in at least three randomly selected fields per section.

### 4.15 Quantification of Inflammatory Cytokines in Serum

To assess the systemic inflammatory response associated with OA progression and treatment, serum levels of pro‐ and anti‐inflammatory cytokines were quantified using ELISA. At the end of the treatment period, whole blood was collected from each rat via cardiac puncture under terminal anesthesia. Blood samples were allowed to clot at room temperature and then centrifuged at 3000 rpm for 10 min to isolate serum.

Commercial ELISA kits were used to determine the concentrations of TNF‐α, IL‐6, IL‐1β, and IL‐10, following the manufacturers' protocols. All assays were performed in duplicate, and absorbance was measured at 450 nm using a microplate reader.

Cytokine concentrations were calculated based on standard curves, and values were expressed as mean ± standard deviation. The cytokine profiles were used to evaluate both the inflammatory status of the OA model and the immunoregulatory effects of the IA microsphere treatments.

### 4.16 Statistical Analysis

All quantitative data were presented as mean ± standard deviation unless otherwise specified. For comparisons among multiple groups, one‐way analysis of variance (ANOVA) followed by Tukey's post hoc test was employed to assess statistical significance. A value of *p* < 0.05 was considered statistically significant, with *p* < 0.01 and *p* < 0.001 indicating higher levels of significance.

All experiments were conducted with a minimum of three biological replicates (n ≥ 3), and all measurements were independently repeated at least three times to ensure reproducibility.

## Conflicts of Interest

The authors declare no conflicts of interest.

## Supporting information




**Supporting File**: advs73851‐sup‐0001‐SuppMat.docx.

## Data Availability

The data that support the findings of this study are available in the supplementary material of this article.
