# Supplementary material for: Injectable Chondroitin Sulfate Methacrylate Hydrogel Microspheres Co‐Loaded with GLPM Nanozyme, Dexamethasone, and Stem Cells for Synergistic Osteoarthritis Therapy
Source: Adv Sci (Weinh). 2026 Jan 30;13(18):e17083. doi: 10.1002/advs.202517083 (PMC13042441; doi:10.1002/advs.202517083)
Supplement: Supplementary file 1 — Supporting File: advs73851‐sup‐0001‐SuppMat.docx. [file ADVS-13-e17083-s001.docx]

Supporting Information

**Injectable Chondroitin Sulfate Methacrylate Hydrogel Microspheres Co-Loaded with GLPM Nanozyme, Dexamethasone, and Stem Cells for Synergistic Osteoarthritis Therapy**

Xiaochen Feng^†^, Ying Fang^†^, Hongwei Yu, Yicheng Wang, Yunze Xu, Chunxiao Shi, Haike Xia, Ranjith Kumar Kankala, Aizheng Chen, Shibin Wang, Chaoping Fu*

Supporting Figures


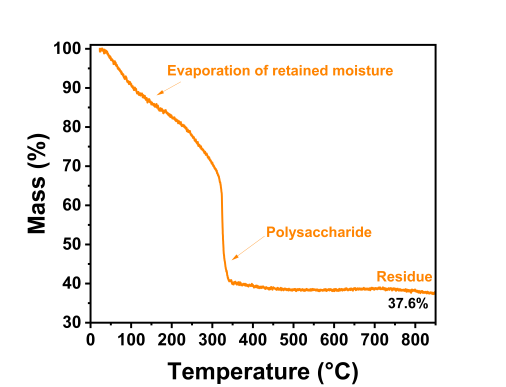


**Figure S1.** Thermogravimetric curve of GLPM.


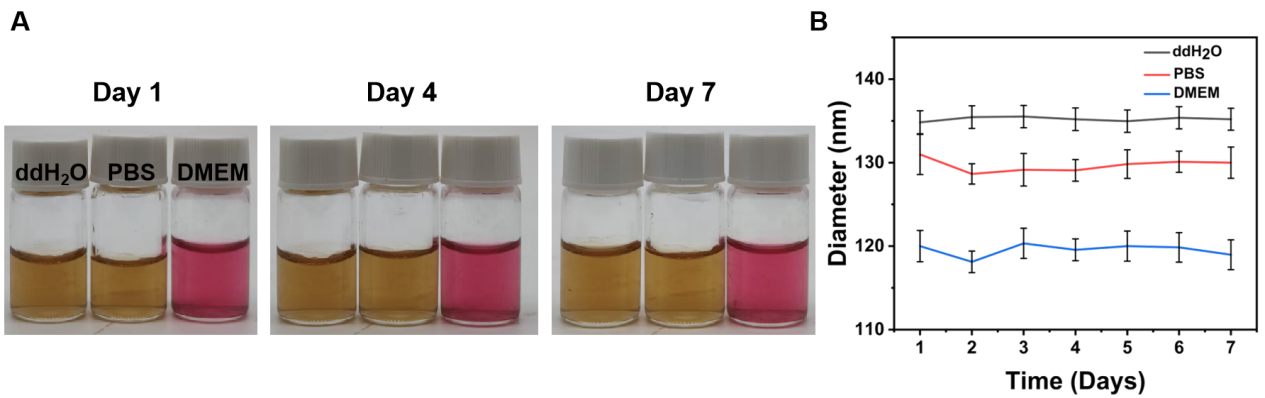


**Figure S2.** Stability characterization of GLPM. (A) Optical photographs of GLPM dispersed in ddH_2_O, PBS, and DMEM on days 1, 4, and 7. (B) Particle size variation curves of GLPM in the above-mentioned media.


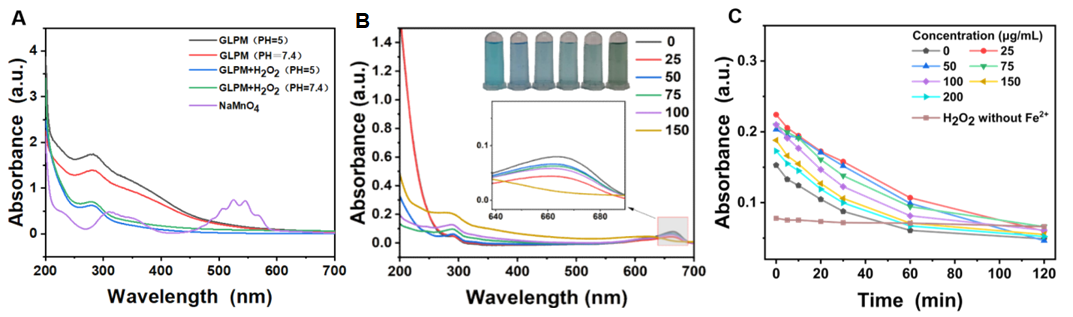


**Figure S3.** *In vitro* characterization of the reactive oxygen species (ROS) scavenging performance of GLPM. (A) UV–vis spectra of GLPM solutions in the presence or absence of H_2_O_2_ under acidic conditions (pH 5.0). (B) Colorimetric changes and UV–vis absorption spectra of methylene blue (MB) after incubation with varying concentrations of GLPM and H_2_O_2_ for 12 h. (C) Absorbance profiles of oxidized TMB products at 652 nm as a function of GLPM concentration and reaction time.


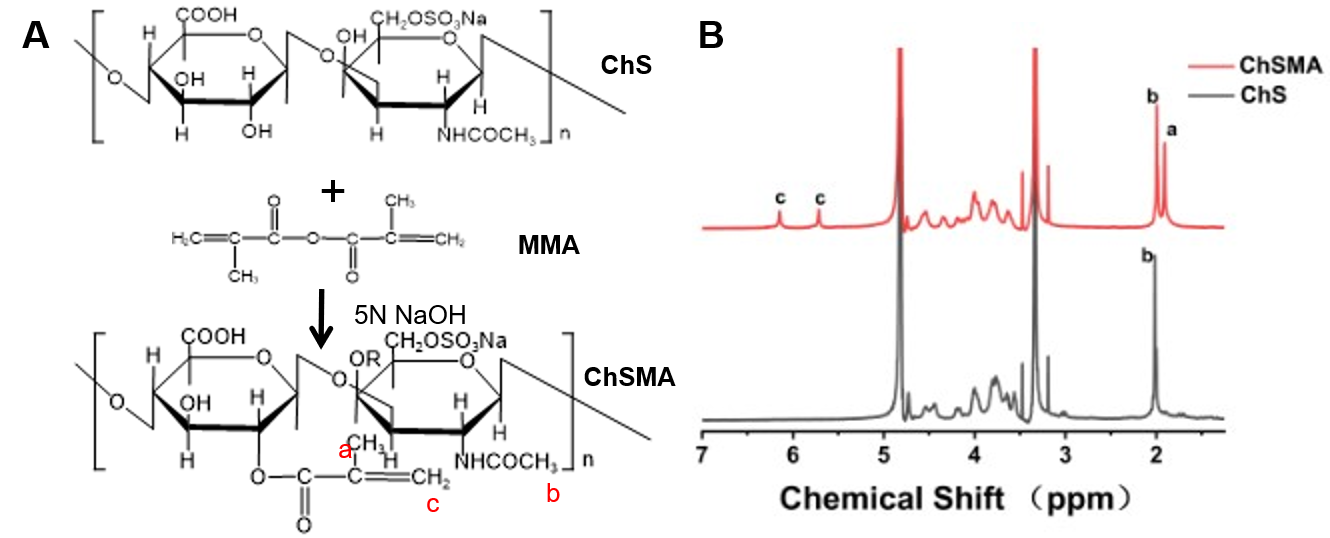


**Figure S4.** Synthesis and characterization of ChSMA. (A) Schematic illustration of the synthetic route for ChSMA. (B) Comparison of 500 MHz 1H NMR spectra between ChS and ChSMA.


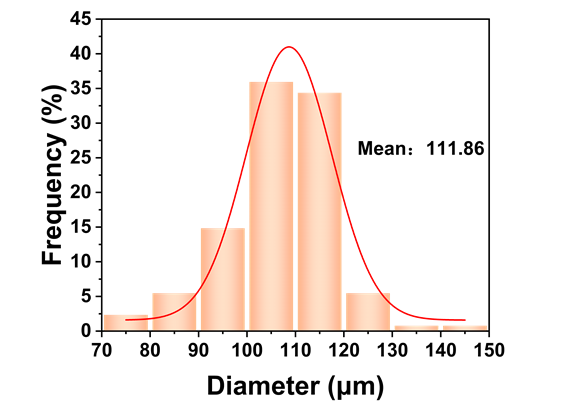


**Figure S5.** Average size distribution of GLPM@ChSMA microspheres measured by optical microscopy and analyzed with ImageJ.


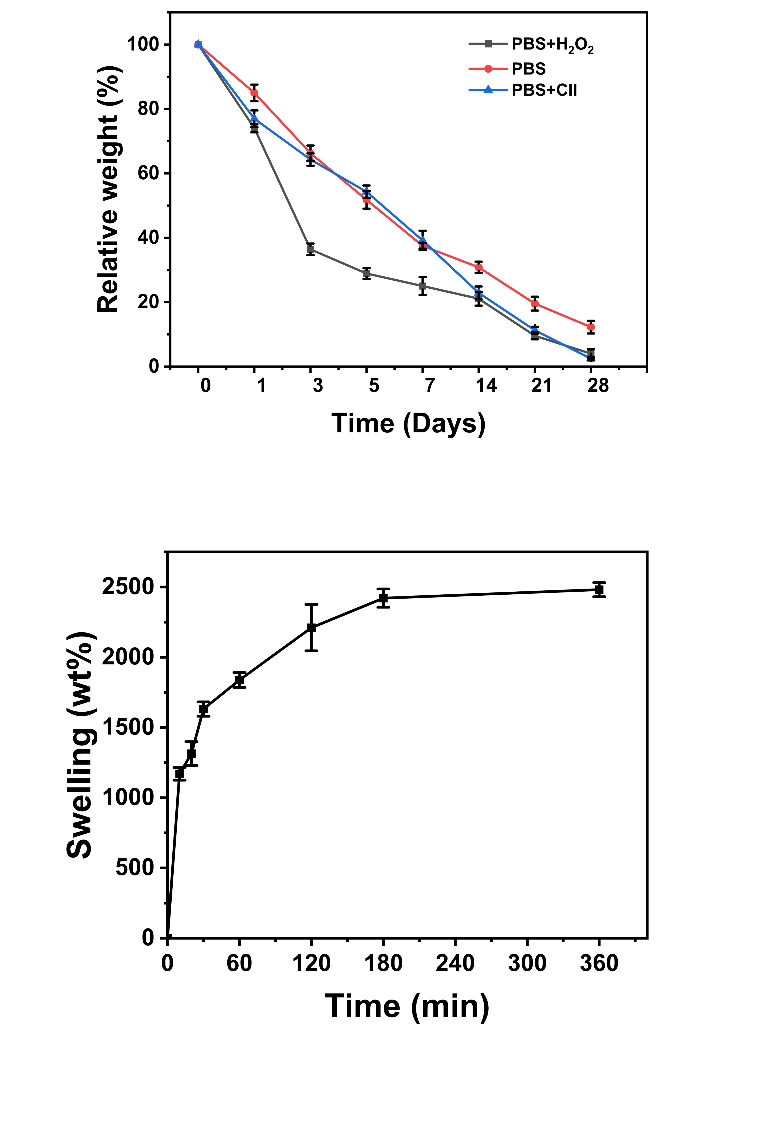


**Figure S6**. Degradation profile of GLPM/Dsp@ChSMA microspheres over 28 days under various conditions.


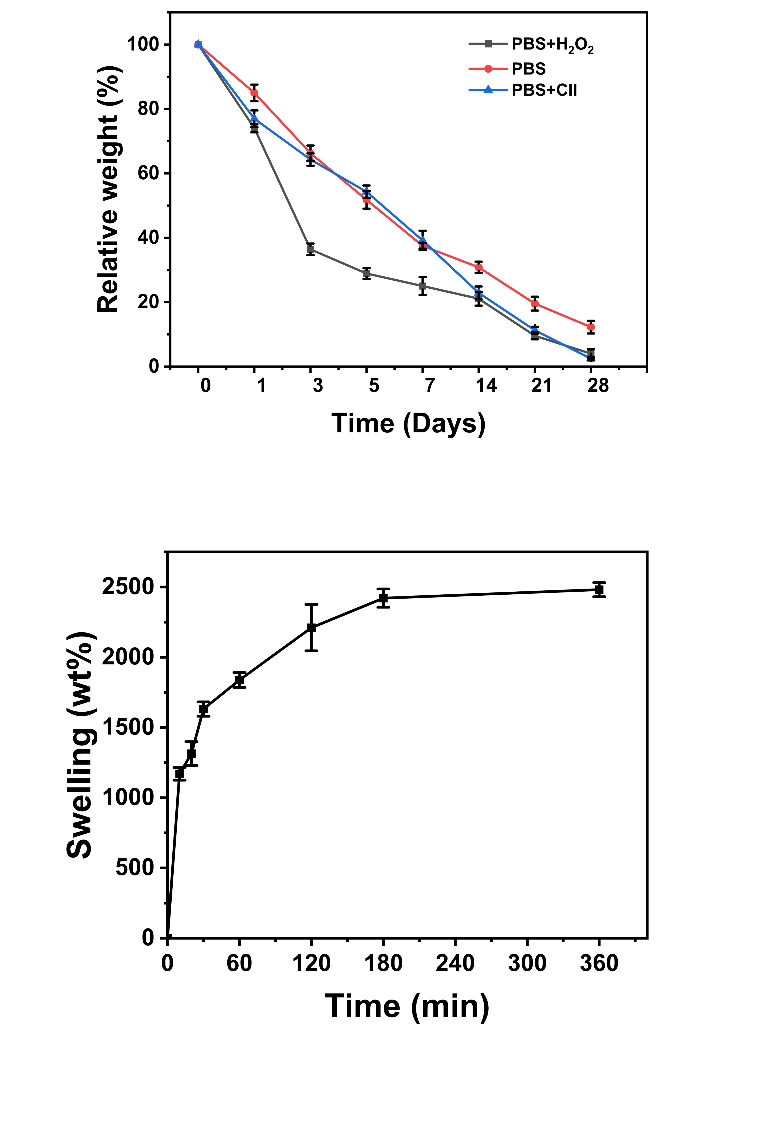


**Figure S7**. Swelling kinetics of microspheres in deionized water.


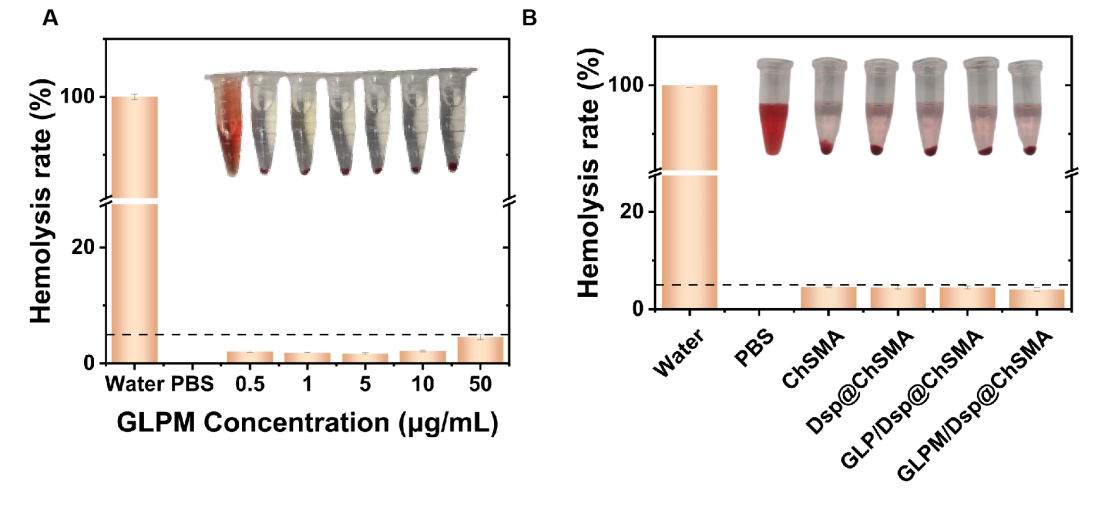


**Figure S8.** Hemolysis rates of GLPM and hydrogel microspheres. (A) Hemolysis rates observed after incubation of red blood cells with GLPM at various concentrations (B) Hemolysis rates measured for hydrogel microspheres, with PBS as the negative control and ultrapure water as the positive control.

**
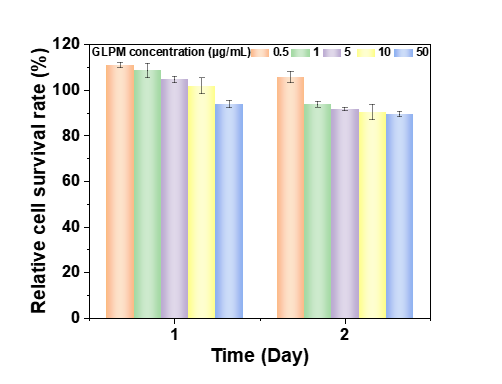
**

**Figure S9.** Cell viability of rat chondrocytes co-cultured with various concentrations of GLPM for 1 and 2 days, assessed by the CCK-8 assay (n = 3).


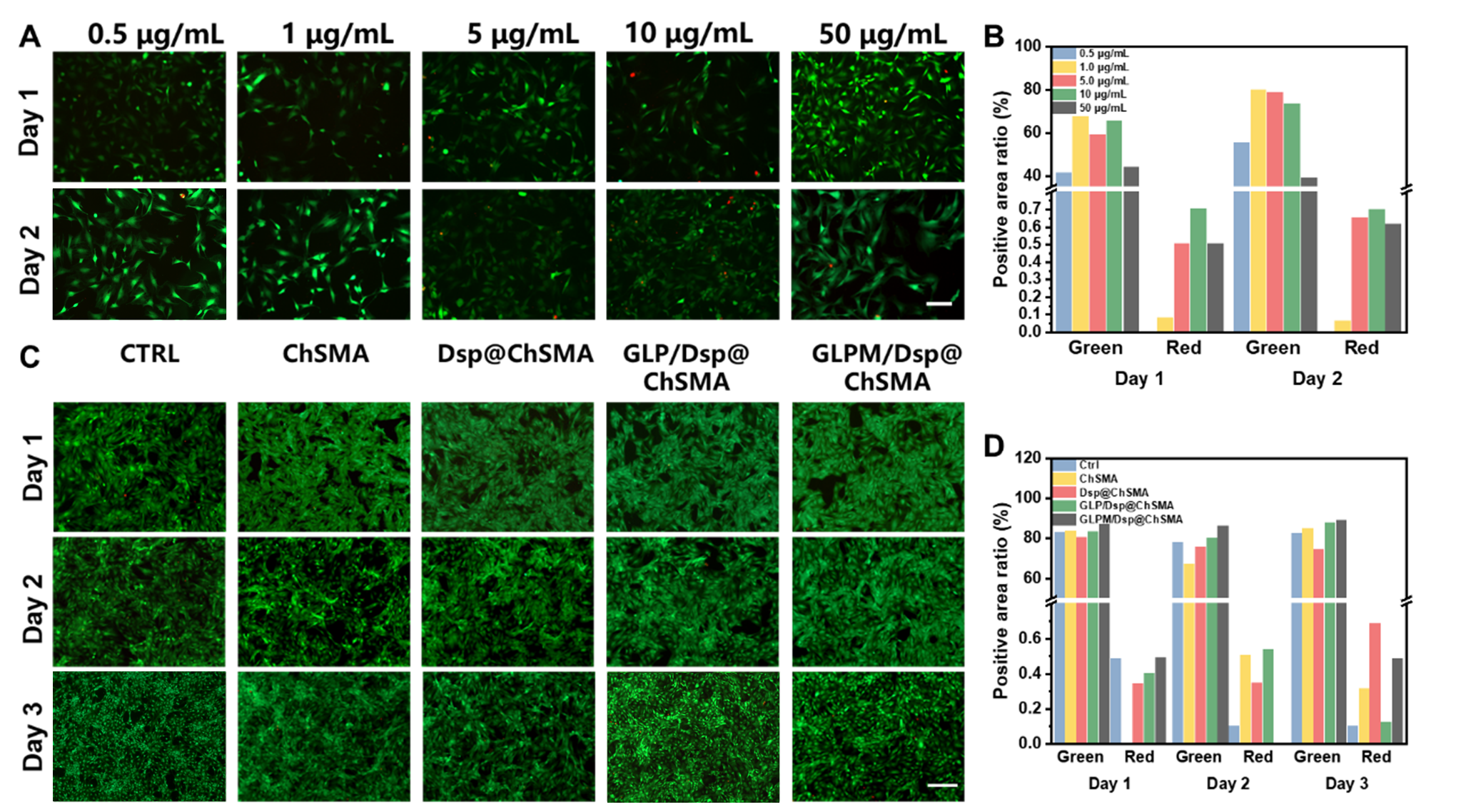


**Figure S10.** Cell viability assessment of rat chondrocytes following exposure to GLPM and microsphere extracts. (A) Calcein-AM/PI staining images of rat chondrocytes co-cultured with varying concentrations of GLPM for 1 and 2 days (scale bar: 200 μm). (B) Quantitative analysis of the fluorescent positive area of chondrocytes after treatment with GLPM for 1 and 2 days. (C) Calcein-AM/PI staining images of rat chondrocytes co-cultured with extracts from microspheres of different compositions on days 1, 2, and 3. (D) Quantitative analysis of the fluorescent positive area of chondrocytes incubated with extracts from microspheres of different compositions on days 1, 2, and 3. Scale bar: 200 μm.


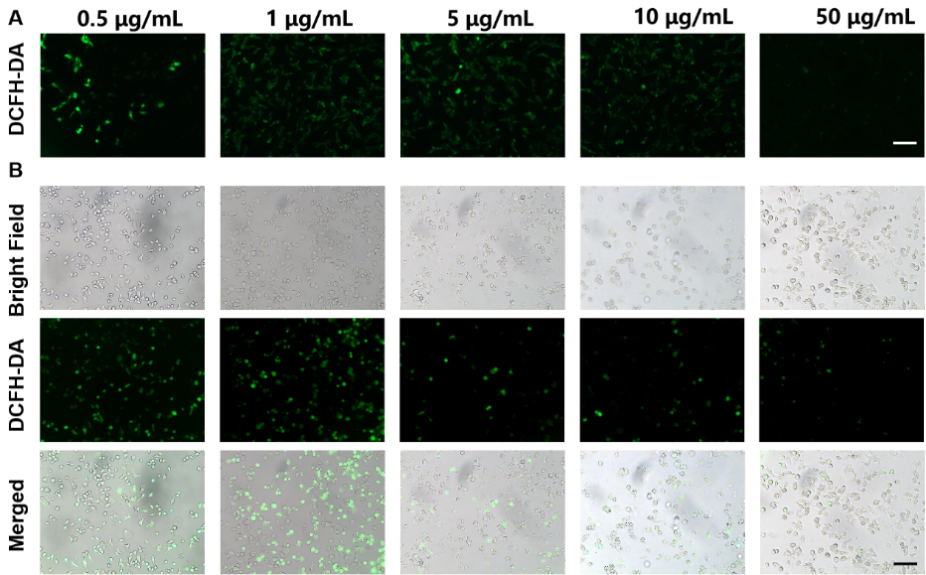


**Figure S11.** Evaluation of the cellular ROS scavenging capability of GLPM. (A) Fluorescence images showing ROS levels in rat chondrocytes after 24 h co-incubation with varying concentrations of GLPM. (B) Fluorescence images illustrating the inhibitory effects of GLPM on LPS-induced intracellular ROS generation in RAW 264.7 cells (scale bar: 200 μm).


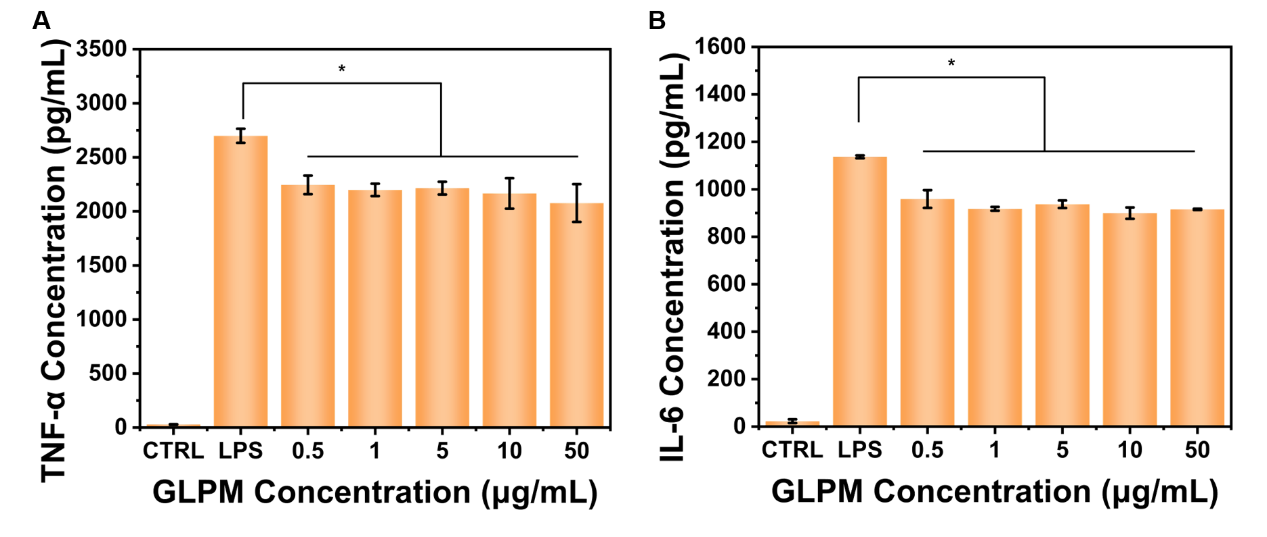


**Figure S12.** Effects of GLPM on LPS-induced inflammatory cytokine secretion in RAW 264.7 cells. (A) Inhibitory effects of GLPM at various concentrations on IL-6 secretion in LPS-stimulated RAW 264.7 cells. (B) Suppression of TNF-α secretion by GLPM treatment (n = 3; *p < 0.05).


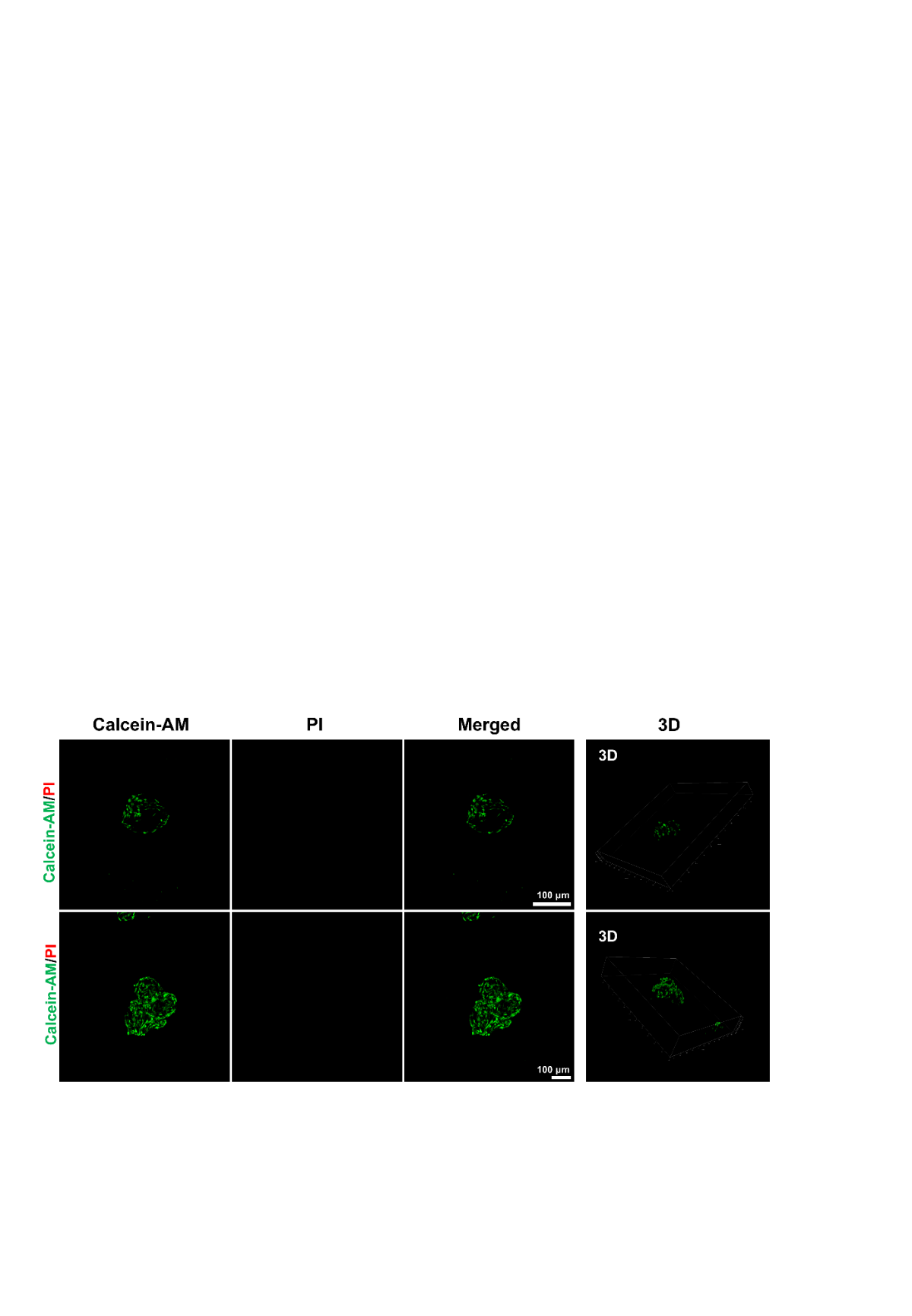


**Figure S13.** Calcein-AM/PI staining and three-dimensional reconstruction of BMSCs after 48 h of co-culture with freeze-dried GLPM/Dsp@ChSMA microspheres.


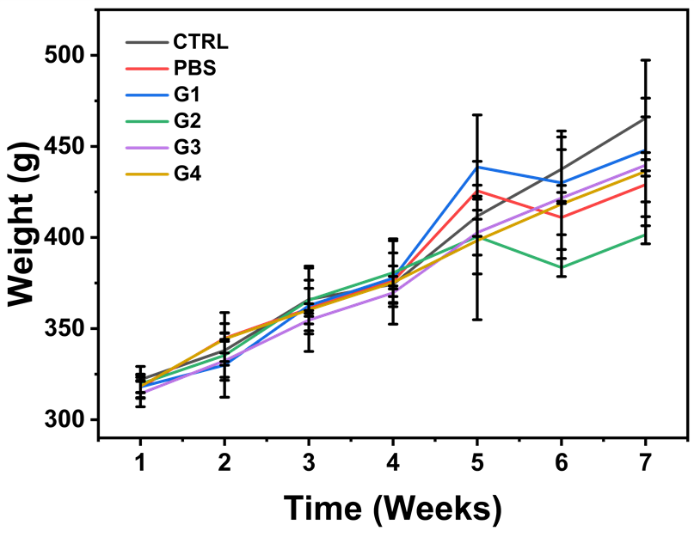


**Figure S14.** Body weight changes of rats in different groups over 7 weeks (n = 6).


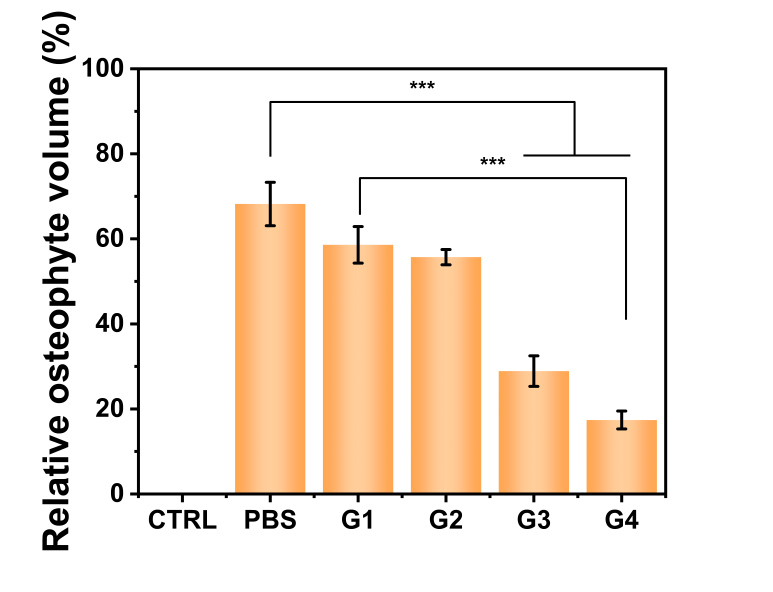


**Figure S15.** Quantitative analysis of total osteophyte volume in knee joints across different treatment groups (*p < 0.05, **p < 0.01, ***p < 0.001).


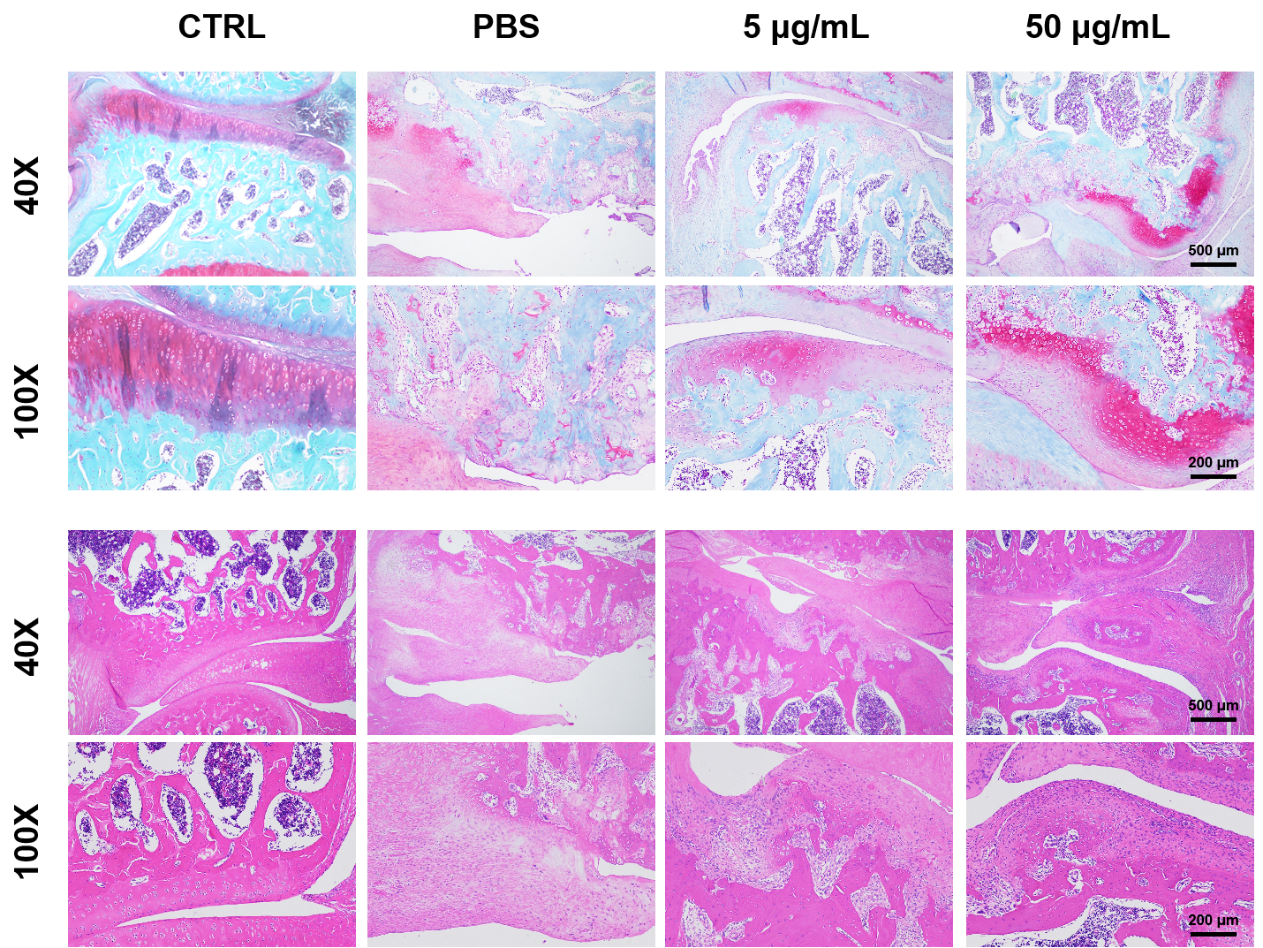


**Figure S16.** Therapeutic effects of GLPM on cartilage repair in rat knee joints. Coronal sections of rat knee joints after 5 weeks of treatment with varying concentrations of GLPM, showing histological analyses using Safranin O–Fast Green staining and H&E staining.


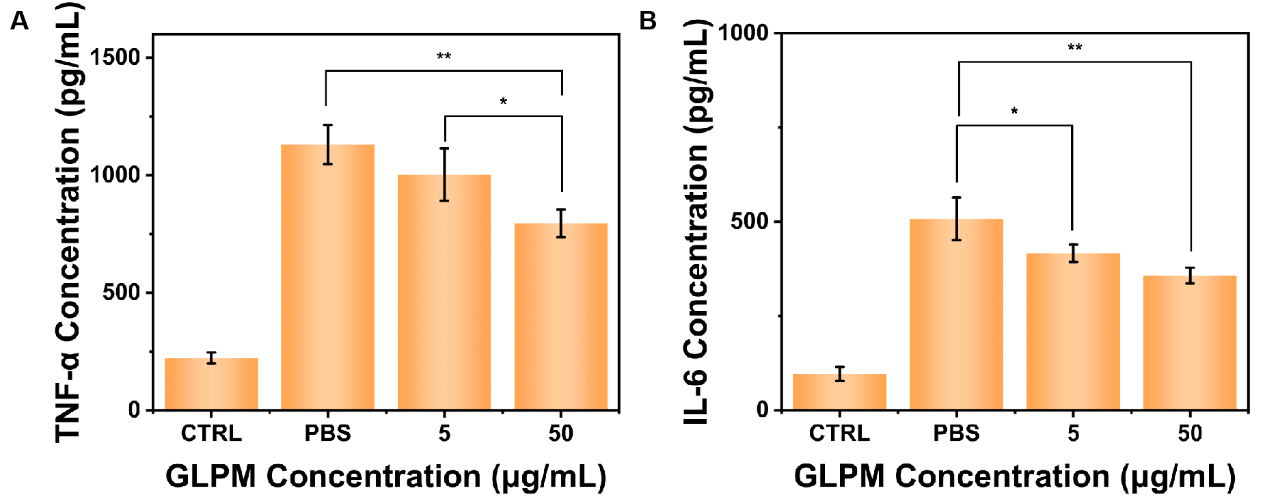


**Figure S17.** Effects of GLPM treatment on serum inflammatory cytokine levels in rats. (A) Changes in TNF-α concentrations in rat serum following 5 weeks of treatment with varying concentrations of GLPM. (B) Changes in IL-6 concentrations in rat serum after 5 weeks of GLPM treatment (n = 3; *p < 0.05, **p < 0.01, ***p < 0.001).

**Figure S18.** Histopathological and biosafety evaluation of GLPM and hydrogel microspheres in rats. (A) Histopathological examination of major organs in rats after 5 weeks of treatment with varying concentrations of GLPM, shown by H&E staining. (B) Biosafety assessment of hydrogel microspheres, illustrated by H&E-stained sections of major organs following 5 weeks of treatment with different microsphere formulations (scale bar: 100 μm).

**
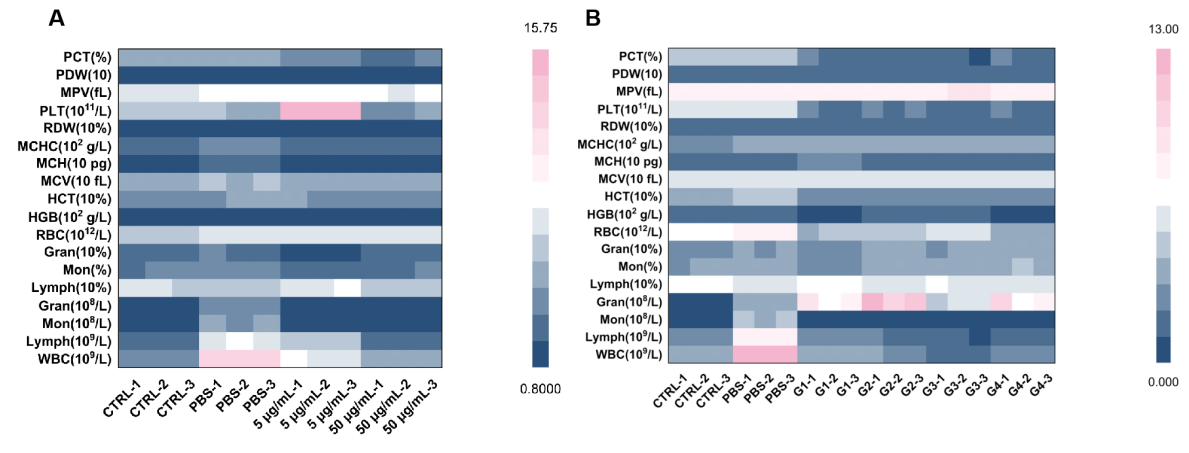
**

**Figure S19.** Hematological parameters of rats following treatment with GLPM and hydrogel microspheres. (A) Blood routine parameters in rats after 5 weeks of treatment with varying concentrations of GLPM. (B) Hematological indices in rats after 5 weeks of treatment with different hydrogel microsphere formulations. For both panels, red indicates an increase and blue indicates a decrease (n = 3).

Supporting Tables

**Table S1.** Comparative summary of recently reported nanozyme-based microsphere systems for OA therapy and the GLPM/Dsp@ChSMA microspheres developed in this study.

| No. | Composition | Particle Size | Cargo Loading | Key Mechanism | Ref. |
| --- | --- | --- | --- | --- | --- |
| 1 | HA-based lubricating microspheres | ~200 μm | NSAIDs or small anti-inflammatory drugs | Nanoparticle-enhanced lubrication; controlled drug release; reduction of cartilage wear | ^[1]^ |
| 2 | SeNP-loaded hydrogel microsphere | 100–130 μm | Selenium nanoparticles | Multistage ROS scavenging; mitochondrial protection; anti-inflammatory signaling | ^[2]^ |
| 3 | HAMA/CS hydrogel microspheres | 200–300 μm | - | Cartilage-like biomechanical support; inflammation inhibition; ECM remodeling | ^[3]^ |
| 4 | PB@HAMA microspheres | ~180 μm | Prussian blue (PB) nanozyme | ROS scavenging; antioxidant protection; inhibition of chondrocyte apoptosis; OA progression attenuation | ^[4]^ |
| 5 | CeO₂-Met@HAMA-DSSe microspheres | 220–230 μm | CeO₂ nanozyme + Metformin | Mitochondrial quality control; ROS scavenging; autophagy activation; cartilage homeostasis maintenance | ^[5]^ |
| 6 | SilMA microspheres containing CaO₂-HAp, BMSCs, macrophages | ~620 μm | CaO₂-HAp + cells | Oxygen release; immune modulation (M2 polarization); osteogenesis | ^[6]^ |
| 7 | BMSCs/GLPM/Dsp@ChSMA microspheres | ~125 µm | Mn nanozyme + Dsp+ BMSCs | ROS scavenging; M2 polarization (HIF-1α activation); Drug anti-inflammation; BMSC paracrine repair | This work |

References

[1] Y. Han, J. Yang, W. Zhao, H. Wang, Y. Sun, Y. Chen, J. Luo, L. Deng, X. Xu, W. Cui, H. Zhang, *Bioact. Mater.* **2021**, *6*, 3596.

[2] J. Liu, J. Liu, S. Liu, P. Xiao, C. Du, J. Zhan, Z. Chen, L. Chen, K. Li, W. Huang, Y. Lei, *Biomaterials* **2025**, *318*, 123195.

[3] Q. Feng, Q. Li, Z. Lin, X. Xu, Y. Huang, H. Dong, X. Cao, *Adv. Healthc. Mater.* **2025**, *14*, 2404877.

[4] W. Peng, J. Lan, M. Goh, M. Du, Z. Chen, *Biomater. Adv.* **2025**, *176*, 214345.

[5] L. Chen, J. Yang, Z. Cai, Y. Huang, Q. Zhang, C. Zhou, J. Wang, W. Huang, W. Cui, N. Hu, *Adv. Healthc. Mater.* **2025**, *14*, 2405069.

[6] A. Deng, H. Zhang, Y. Hu, J. Li, J. Wang, X. Chen, Z. Geng, K. Xu, Y. Lai, J. Wang, Y. Jing, L. Bai, J. Su, *Adv. Sci.* **2025**, e01437.
